# Mitigating Early Phosphatidylserine Exposure in a Tmem30a‐Dependent Way Ameliorates Neuronal Damages After Ischemic Stroke

**DOI:** 10.1002/mco2.70140

**Published:** 2025-03-18

**Authors:** Chuanjie Wu, Jiaqi Guo, Yunxia Duan, Jiachen He, Shuaili Xu, Guiyou Liu, Chen Zhou, Yuchuan Ding, Xianjun Zhu, Xunming Ji, Di Wu

**Affiliations:** ^1^ Department of Neurology and China‐America Institute of Neuroscience Beijing Institute of Geriatrics Xuanwu Hospital Capital Medical University Beijing China; ^2^ Beijing Key Laboratory of Hypoxia Conditioning Translational Medicine Beijing China; ^3^ Center of Stroke Beijing Institute for Brain Disorders Capital Medical University Beijing China; ^4^ Department of Neurosurgery Wayne State University School of Medicine Detroit Michigan USA; ^5^ The Sichuan Provincial Key Laboratory for Human Disease Gene Study and Department of Laboratory Medicine Center for Medical Genetics Sichuan Provincial People's Hospital University of Electronic Science and Technology of China Chengdu Sichuan China

**Keywords:** Annexin V, ischemic stroke, neuroprotection, penumbra, phosphatidylserine, Tmem30a

## Abstract

Phosphatidylserine (PS) exposes to the outer plasma membrane after a pathological insult (e.g., stroke) but not under normal conditions whereby PS remains within the inner plasma membrane. However, the reversibility and translational potential of PS exposure in damaged cells after stroke are still unknown. Here, we demonstrated that plasma Annexin V, which has a high affinity to membranes bearing PS, was increased in patients with salvage penumbra after endovascular therapy, and associated with early neurological improvement. Moreover, Annexin V treatment could decrease PS exposure and mitigate neurological impairments in transient ischemia/reperfusion mouse models, but not in permanent ischemia. Furthermore, we used a combination of cell, rodent, and nonhuman primate ischemia/reperfusion models and found that transmembrane protein 30A (Tmem30a) was increased in the ischemic penumbra after stroke and imperative for less PS exposure and better neurological functions. Mechanistically, mitigation of PS exposure mediated by Tmem30a/Annexin V connection led to decreased expression of apoptosis and necroptosis markers in neurons of penumbra. Overall, our findings reveal a previously unappreciated role of reducing PS exposure by Annexin V treatment in protecting the penumbra in a clinically relevant ischemia/reperfusion model. Tmem30a is essential for reducing PS exposure in the penumbra after ischemic stroke.

## Graphical abstract

The ischemic penumbra is the hypo‐perfused brain tissue at risk of progressing to infarction but is still salvageable if re‐perfused. Wu et al. demonstrate increased plasma Annexin V reduced PS exposure in a Tmem30a‐dependent way in the penumbra after stroke, consequently leading to a better neurological outcome. Thus, Tmem30a‐mediated externalization of PS may be a novel mechanism and target in stroke.

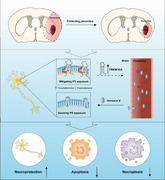

## 1 Introduction

The brain has a remarkable capacity for self‐protection, manifested by transient ischemia, preconditioning, and exercise‐induced protective responses [1]. The ischemic penumbra is the hypo‐perfused brain tissue at risk of progressing to infarction, but still salvageable if reperfusion therapy is achieved [2]. Emerging research indicates that neuronal exposure to stressors like focal ischemia hypoxic conditions initiates endogenous neuroprotective mechanisms, activating sequential survival signaling pathways to prevent cellular apoptosis [3]. For instance, during focal ischemia, neurons sent “help‐me” signals, and astrocytes subsequently released functioning mitochondria to assist the stressed neurons [4]. However, ischemic penumbra is still at great risk of progressing to infarction if untreated. As a groundbreaking advancement in acute stroke management, endovascular therapy (EVT) effectively overcomes cerebral perfusion deficits through timely revascularization, fundamentally transforming therapeutic outcomes in ischemic brain disease [5, 6, 7]. Thus, these “help‐me” signals, which had previously been overlooked or failed to be translated due to lack of consideration of recanalization therapy, may be novel targets for clinical translation [8]. We hypothesize that these signals from penumbra tissue might be the translational targets for neuroprotection in stroke and will contribute to robust cytoprotective effects in preclinical models in the era of recanalization.

Phosphatidylserine (PS), which has long been identified as a distinctive characteristic of cell death, was exposed to the outer plasma membrane. PS is typically maintained in the inner lobules of the plasma membrane under normal conditions [9]. Increasing evidence demonstrates that PS acts as “eat‐me” signals on the cell surface of these still viable cells when exposed to harmful stimuli [10]. Previous studies have found that LPS‐induced neuronal death was significantly reduced by blocking PS recognition with Annexin V and the anti‐PS antibody, but few of these findings have been reported in vivo model [11]. Recent study found that recombinant Annexin V could cross blood‐brain barrier (BBB) and reduce PS exposure in the inhibitory post‐synapses and mitigate abnormal excitability and seizures [12]. Thus, we hypothesized that PS exposure might be observed in the penumbra tissue after ischemic stroke and reducing PS exposure may be an attractive translational approach to protecting penumbra and mitigating ischemic damages.

Transmembrane protein 30A (*Tmem30a*) acts as a flippase, transporting aminophospholipids (mostly PS) from the plasma membrane's outer leaflets to its inner leaflets [13]. *Tmem30a* preserves a steady lipid raft, which is a conserved function in both humans and mice [14, 15]. As reported, *Tmem30a* can recruit P4‐ATPase ATP8A1 to translocate PS inwardly and overexpression of *Tmem30a* can greatly enhanced cell migration in hamster ovary cells [16]. Recent research has emphasized the significance of *Tmem30a* in vivo. *Tmem30a*, for instance, was necessary for the survival of retinal photoreceptors in the retina [17]. In mature neurons, acute deletion of Tmem30a resulted in selective loss of inhibitory postsynapses and preferential PS exposure in neuronal somas, with no effect on other synapses [12]. More importantly, Tmem30a function is closely associated with energy status. Ischemia/reperfusion process will have a notable effect on Tmem30a. Thus, we hypothesized that *Tmem30a* plays an important role in reducing PS exposure and maintaining cell membrane stability in the penumbra tissue after focal ischemia.

Annexin V, with a high affinity to membranes bearing PS, had previously been used to combine with fluorescent label to test cell death. Moreover, plasma Annexin V level was significantly increased in patients with acute coronary syndrome [18]. However, changes and functions of Annexin V are less reported in stroke.

Emerging research has extensively documented crosstalk linking peripheral organ systems with the central nervous system (CNS). Contemporary investigations have revealed that numerous blood‐borne signaling molecules—including both novel discoveries and previously understudied compounds—function as critical mediators in orchestrating these systemic interactions [19]. Thus, we hypothesized that Annexin V might be a clinical translational target in the penumbra protection after stroke. We would also explore underlying mechanisms.

## 2 Results

### 2.1 Increased Plasma Annexin V Levels Are Associated With Neurological Benefits in Patients Exhibiting the Penumbra

**FIGURE 1:**
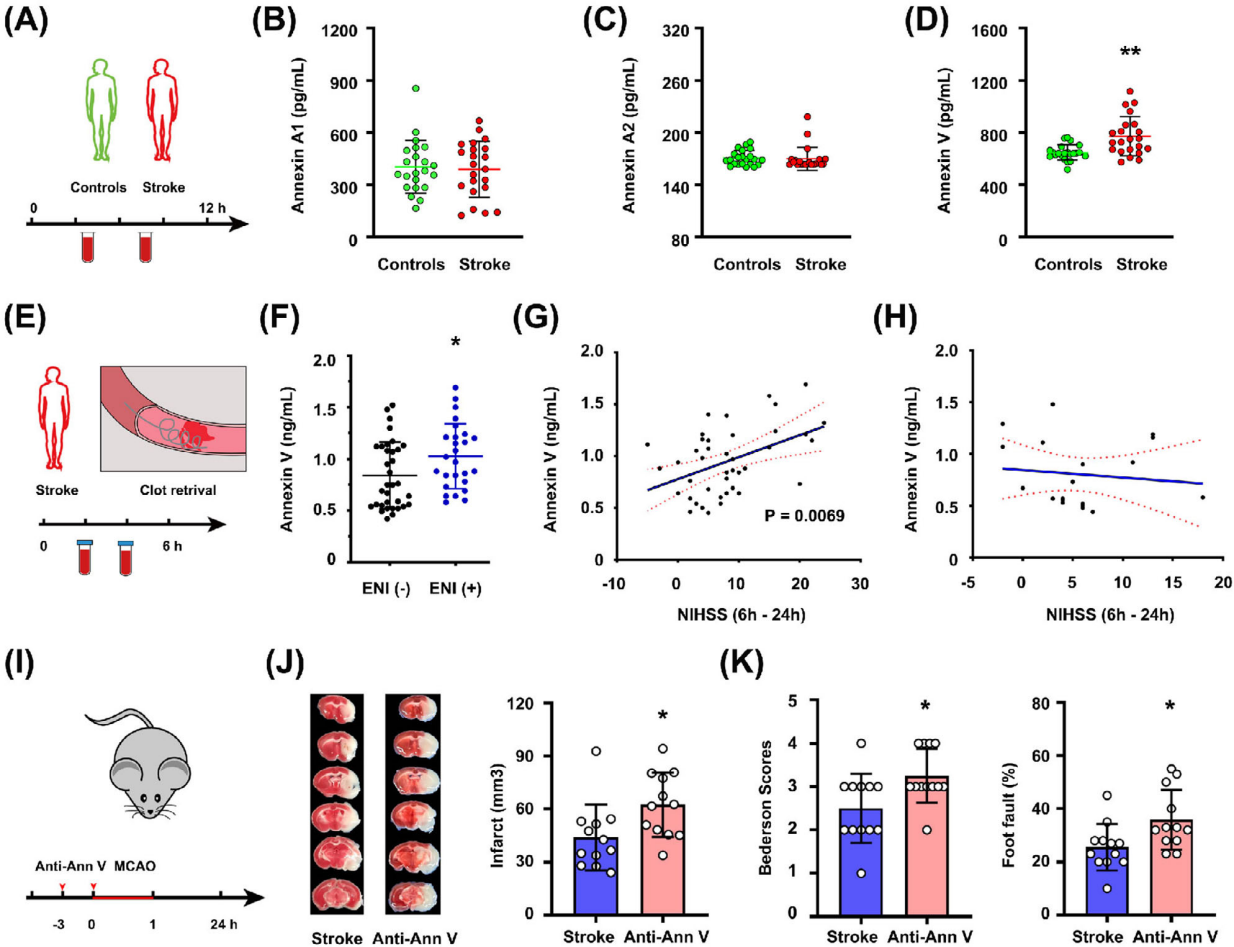
Plasma Annexin V levels increased after stroke and associated with better outcome. (A) Schematic of blood collection in stroke patients. (B–D) Annexin V levels were increased in stroke patients. **p < 0.01, two‐tailed *t* test. (E) Schematic of blood collection in stroke patients receiving EVT. (F) Comparison of Annexin V plasma levels in acute ischemic patients after EVT with or without ENI. *p < 0.05, two‐tailed *t* test. (G and H) Linear regression analysis correlating a better NIHSS improvement and increasing Annexin V concentrations in patients with penumbra at baseline (*r* = 0.488; *p* = 0.0069), but not in patients without penumbra at baseline. (I) Experimental paradigm of Annexin V antibody injection in tMCAO (transient MCA occlusion) mice (*n* = 12). (J) Representative images and quantification of TTC staining in tMCAO (transient MCA occlusion) mice receiving Annexin V antibody after stroke. *p < 0.05, two‐tailed *t* test. (K) Neurologic scores in tMCAO (transient MCA occlusion) mice. *p < 0.05, two‐tailed *t* test.

First, we used a case–control gene expression dataset from GEO (GSE58294) to evaluate the potential differential mRNA expression of Annexin V in 69 cardioembolic stroke samples and 23 normal control samples. We found that Annexin V expression levels were 1.525, 1.485, and 1.714 times higher than those in control samples at less than 3, 5, and 24 h after ischemic onset, respectively, by using an online analysis tool GEO2R (https://www.ncbi.nlm.nih.gov/geo/geo2r/) [20] (Table S1). Then, healthy controls (*n* = 22) and stroke patients (*n* = 22) with first episode of ischemic stroke and presentation within 12 h of onset of symptoms were included (Figure 1A, demographic data in Table S2). We found that Annexin A1 and Annexin A2 concentrations were no significant differences in stroke patients (Figure 1B, C). However, plasma Annexin V levels were higher in stroke patients when compared to healthy controls (Figure 1D).

Then, we investigated if early neurological improvement (ENI) in acute ischemia individuals was linked to a greater plasma Annexin V level. In patients undergoing EVT, ENI was previously characterized as a decline of 8 or more points on the National Institutes of Health Stroke Scale (NIHSS) or an NIHSS score of 0 or 1 at 24 h (Figure 1E, F) [21, 22]. We consecutively enrolled a total of 60 patients receiving EVT within 6 h after symptom onset. These patients' initial clinical and demographic traits were similar and presented in Table S3. We found that plasma Annexin V concentrations in patients with ENI (*n* = 26) were higher than those in patients without ENI (*n* = 34) (mean 1.02 vs. 0.83, *p* = 0.028). Next, we analyzed the effect of penumbra existence on the association between plasma Annexin V concentrations and NIHSS change between baseline and 24 h (△NIHSS). A positive correlation was found between high Annexin V concentrations and △NIHSS (*p* = 0.0069) in patients exhibiting penumbra (*n* = 41) based on computer tomography perfusion imaging or magnetic resonance perfusion (Figure 1G). However, we did not find the correlation in patients without penumbra (*n* = 19; *p* = 0.63) (Figure 1H). Thus, a higher Annexin V level might be associated with a better outcome in stroke patients exhibiting penumbra after EVT.

Lastly, we assessed whether specific Annexin V inhibition by monoclonal antibody could affect stroke outcomes in a mouse model of stroke. In normal mice, anti‐Annexin V antibody (40 µg/kg) reduced plasma Annexin V approximately 40% after 3 h administration (Figure S1A). When compared to control mice following 60 min of middle cerebral artery occlusion (MCAO), mice injected with anti‐Annexin V antibody prior to ischemia experienced a greater infarction and a worse neurologic deficit 24 h after stroke onset (Figure 1I–K). Moreover, TUNEL+ cells were increased in penumbra areas in mice receiving anti‐Annexin V 24 h after stroke, as compared to those receiving vehicle (Figure S1B, C).

Collectively, these results suggested that a higher Annexin V concentration might be beneficial to stroke outcomes.

### 2.2 Annexin V Inhibits PS Exposure and Exert Benefits Only in Transient MCAO Models

According to reports, recombinant Annexin V can cross the BBB and prevent the exposed PS from attaching to its binding partners [12, 23]. Thus, we hypothesized that preventing PS exposure might be feasible to attenuate ischemic damages after stroke.

**FIGURE 2:**
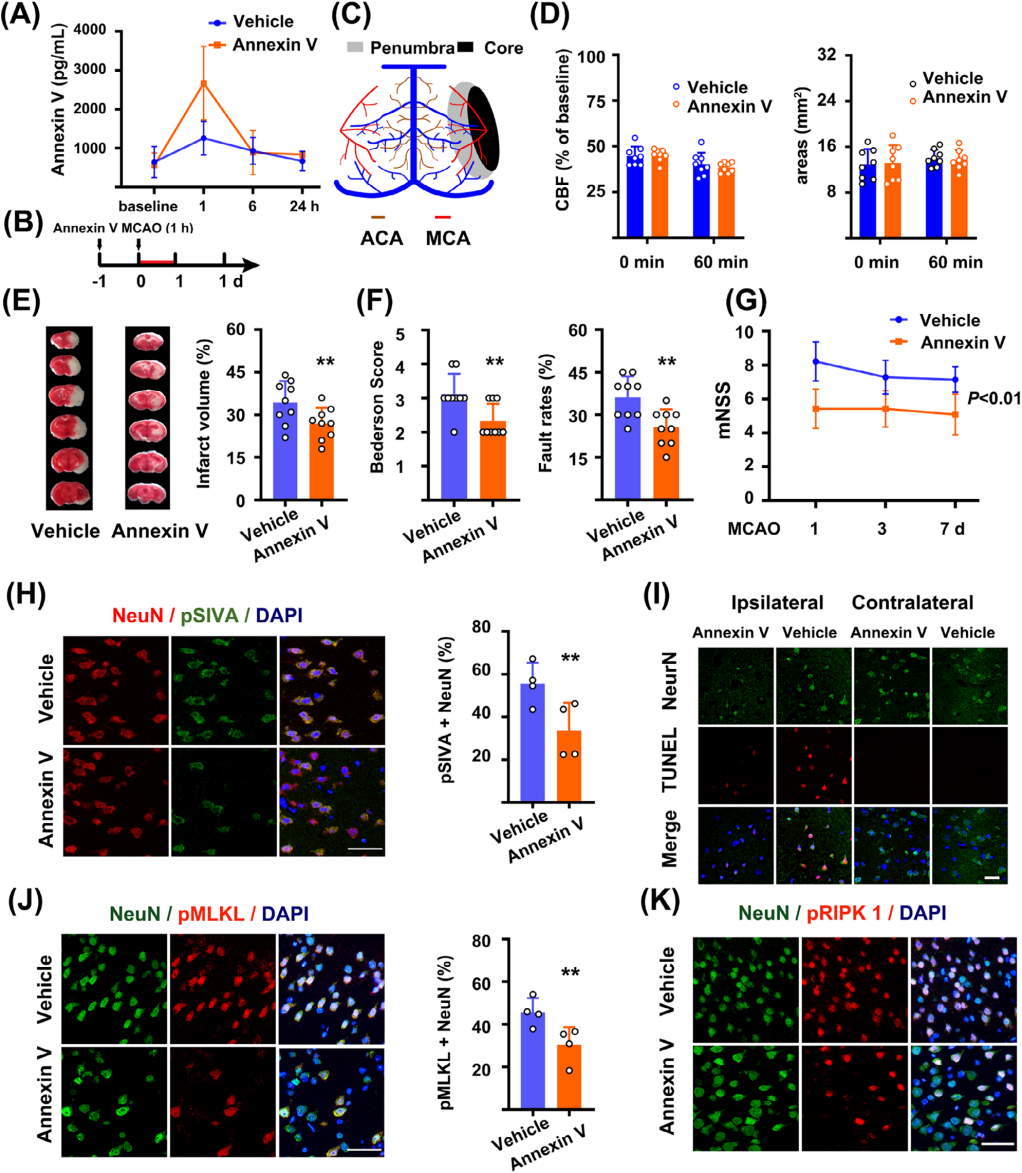
PS blocking reduces brain damages after ischemia/reperfusion. (A) Plasma Annexin V levels after intravenous injection in WT mice (*n* = 6). (B) Schematic diagram of the experiment in which recombinant Annexin V was injected intravenously in wild‐type C57BL/6 mice. (C) Schematic drawing of the LSI view on pial vessels and regions of interest (ROIs) during focal ischemia in mice. (D) LSI recordings for CBF levels and penumbra areas in tMCAO (transient MCA occlusion) mice receiving vehicle or Annexin V (*n* = 9). (E) Representative TTC‐stained brain sections and infarct volumes 24 h after tMCAO (transient MCA occlusion) (*n* = 9). **p < 0.01, two‐tailed *t* test. (F) Neurological scores in tMCAO (transient MCA occlusion) mice 1 day after stroke (*n* = 9). **p < 0.01, two‐tailed *t* test. (G) mNSS in Annexin V‐treated and control mice 7 days after stroke (*n* = 6). **p < 0.01, two‐tailed *t* test. (H) Representative images and quantification of pSIVA^+^ (PS exposure) neuron in penumbra regions 24 h after 60min tMCAO (transient MCA occlusion) (*n* = 4). Bar = 10 µm. (I) Representative confocal images of neuronal death based on a neural marker NeuN and TUNEL assay in the ischemic ipsilateral brain regions of mice 24 h after 60 min tMCAO (transient MCA occlusion) (*n* = 4). Bar = 50 µm. (J) Representative images and quantification of pMLKL^+^ neurons in penumbra regions 24 h after 60 min tMCAO (transient MCA occlusion) (*n* = 4). Bar = 10 µm. (K) Representative images of pRIPK1^+^ neurons in penumbra regions 1 day after tMCAO (transient MCA occlusion) (*n* = 4). Bar = 10 µm.

We assessed whether recombinant Annexin V could affect stroke outcomes in a mouse model of stroke. In normal mice, plasma Annexin V levels rose sharply 60 min after intravenous injection (Figure 2A). Annexin V levels in the brains remained unchanged, but rose to a higher level than those receiving vehicle at 24 h after injection (Figure S2A). We intravenously injected Annexin V twice [12], then subjected animals to transient MCAO (Figure 2B). There were no significant differences in the core and penumbra regions between the two groups at time points 0 and 60 min after stroke based on laser speckle imaging (LSI) recordings (Figure 2C, D, Figure S2B–D). Annexin V administration reduced infarct sizes 24 h after 60min MCAO (Figure 2E). Consistent with the reduced infarct sizes, neurological scores were also improved both at 24 h and throughout 7 days in mice receiving Annexin V (Figure 2F, G). More importantly, pSIVA^+^(PS exposure) neurons in penumbra areas were reduced in mice receiving Annexin V administration (Figure 2H). Apoptosis and necroptosis markers were reduced in penumbra areas in mice receiving Annexin V 24 h after stroke, as compared to those receiving vehicle, including TUNEL^+^, pMLKL^+^, and pRIPK1^+^ neurons (Figure 2I–K, Figure S2E, F).

The penumbra is the conceptual target for reperfusion‐therapy in ischemic stroke. However, the penumbra will collapse and turn into the infarct when focal ischemia continues. Thus, to test the potential benefits of Annexin V on the penumbra, we performed the same procedure of Annexin V administration in a mice model of permanent occlusion as a negative control. In contrast to the transient MCAO models, there were no differences in both infarct sizes (Figure S2G) and neurological score between the two groups (Figure S2H). Thus, these data indicated that recombinant Annexin V crossed the BBB, targeted the penumbra, and conferred neurological benefits in ischemia/reperfusion models after stroke.

### 2.3 *Tmem30a* is Upregulated in the Penumbra Region Shortly After Ischemic Stroke

**FIGURE 3:**
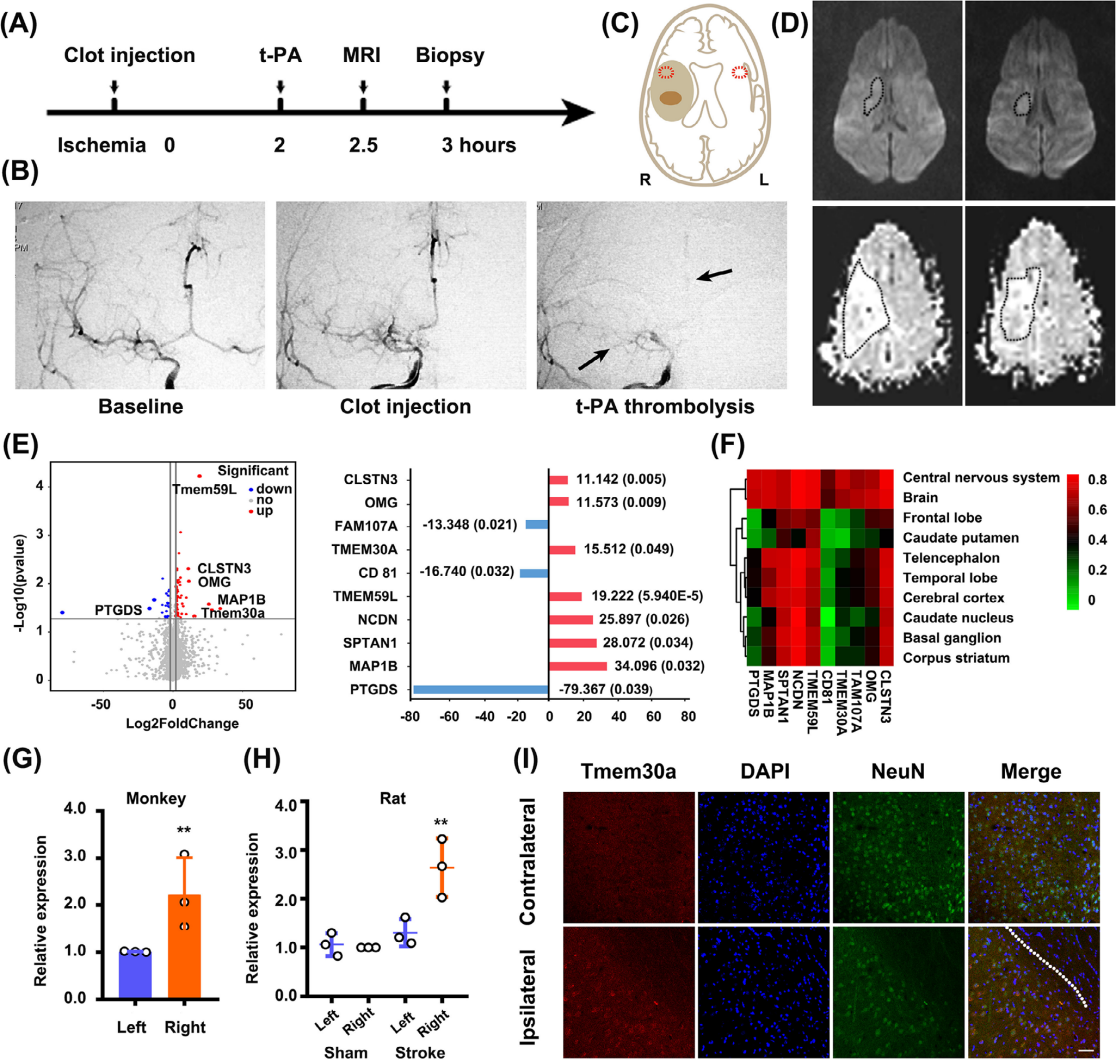
Tmem30a is upregulated in neurons within the penumbra tissues. (A) The schematic diagram exhibits ischemic induction, t‐PA thrombolysis, MRI scans, and biopsy in adult rhesus monkeys (*n* = 3). (B) Digital subtraction angiography of blood perfusion in the baseline, post clot‐injection, and after thrombolysis. Arrow stands for the occluded MCA and ACA after thrombolysis. (C) Schematic images of the penumbra in light brown, the core in dark brown, and the biopsy site in red. (D) DWI (up) and PWI images (down) after stroke. (E) Analysis of differential gene expression was based on RNA‐seq and presented as a volcano plot in the penumbra tissue of rhesus monkeys. The top 10 upregulated (in red) and downregulated (in blue) genes based on adjusted *p* value are listed. (F) The heatmap in R with the heatmap package indicates that Tmem30a transcript is expressed in the brain, but generally at lower levels in the cerebral cortex. (G and H) qRT‐PCR analysis of Tmem30a expression in the penumbra and the corresponding area in the contralateral side in monkey (*n* = 3) (G) and rat tMCAO (transient MCA occlusion) models (*n* = 3) (H). (I) Immunofluorescence staining of Tmem30a positive neurons in the cerebral penumbra cortex 24 h in rat tMCAO (transient MCA occlusion) models. Core in the upside of the dash‐line, penumbra in the downside. Bar = 50 µm. DAPI, 4′ 6‐diamidino‐2‐phenylindole. **p < 0.01, two‐tailed *t* test for (G), and by one‐way ANOVA with Bonferroni's multiple comparison in (H)

Due to the contrast results of Annexin V in ischemia/reperfusion and permanent ischemia, we hypothesized that potential mechanisms might derive from the penumbra. We previously established a thrombus and thrombolysis model in rhesus monkeys [24, 25]. In brief, MCA occlusion was confirmed radiographically by digital subtraction angiography after the clot injection (Figure 3A, B, arrow). Based on widely accepted definition of penumbra in stroke models and patients [26, 27], the mismatch between perfusion and diffusion‐weighted MR images (PWI/DWI mismatch), we got the penumbra tissues and their normal counterpart tissues at approximately 3 h after ischemic onset (Figure 3C, D, Figure S3A). We confirmed the biopsy sites for penumbra by another definition, which is the presence of a DWI‐fluid‐attenuated inversion recovery (FLAIR) mismatch in patient selection (Figure S3B) [28].

We made a comparison of transcriptional profiling between penumbra tissues (3 h after stroke) in the ipsilateral and contralateral hemisphere to identify individual differences in the brain tissues potentially underlying acute ischemic responses. RNA‐seq analysis found that a total of 89 mRNAs (23 down and 66 up) changed dramatically with a value of log 2 (fold change) > 1.5. Among the top 10 differentially expressed mRNAs (Figure 3E), four of them were expressed at low level in normal tissue (Figure 3F, https://hb.flatironinstitute.org/gene), in which only *Tmem30a* and *OMG* were significantly upregulated after focal ischemia. Gene ontology analysis showed that regulation of biological quality, localization, and signaling were ranked as the top three most important factors in biological process analysis; protein binding was the most significant factor in the molecular function analysis; and plasma part, neuron part, and cell projection as the top three important factors in the cellular component analysis (Figure S4A–C). As a β‐subunit of P4‐type ATPase, *Tmem30a* is essential for the maintenance of asymmetric distribution of phospholipids. Thus, we speculated that *Tmem30a* might act as a signal to preserve neuron in the primary stage of focal ischemia.

Real‐time PCR (RT‐PCR) further showed an increased expression of *Tmem30a* in the penumbra of the ipsilateral hemisphere in both monkey and rat stroke models (Figure 3G, H). Moreover, *Tmem30a* co‐localized with NeuN^+^ neurons in the penumbra tissues (Figure 3I), while control images from the opposite hemisphere exhibited less double staining neurons (Figure S4D). Additionally, we did not observe the co‐localization of *Tmem30a* with GFAP^+^ astrocytes or Iba1^+^ microglia in the penumbra tissues (Figure S4E). These data indicated that *Tmem30a* were upregulated in neurons within the penumbra in two stroke models.

### 2.4 *Tmem30a* Upregulation is Necessary for Less PS Exposure and Neural Benefits in Oxygen Glucose Deprivation/Reoxygenation Models

**FIGURE 4:**
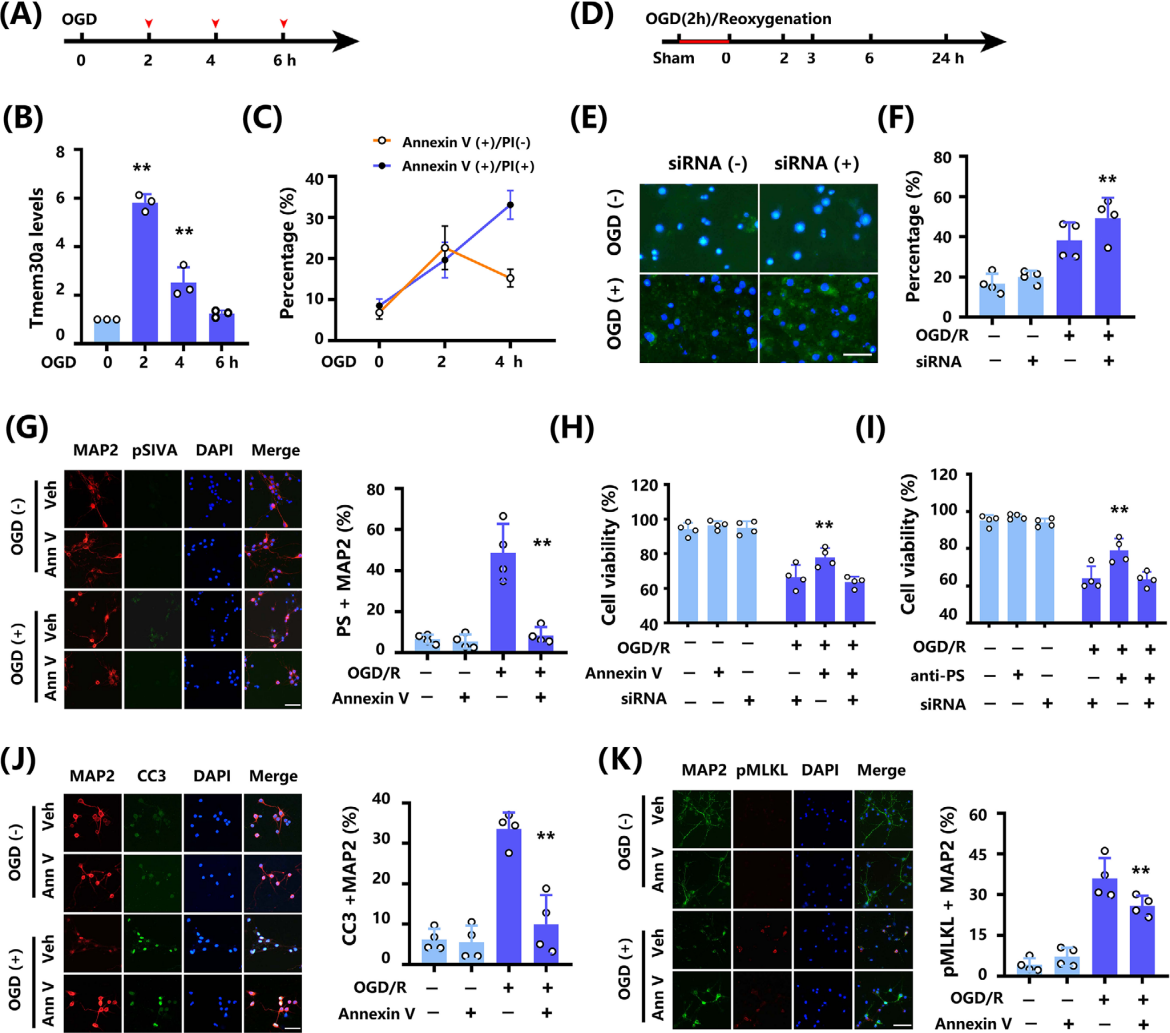
Upregulation of Tmem30a in neuron mitigates PS exposure and cell death. (A and B) Diagram exhibiting effects of OGD tests with various durations on *Tmem30a* levels and PS exposure (*n* = 3). (C) Percentage of Annexin V− FITC positive/PI negative or positive cells after OGD. (D) Diagram showing 2 h OGD and with reoxygenation for 0, 2, 3, 6, and 24 h. (E and F) Representative images and quantification of Annexin V− FITC positive after 2h OGD and 3h reoxygenation (*n* = 4). (G) Representative images and quantification of pSIVA positive (PS exposure) cells in primary neuron undergoing 2h OGD and 24h reoxygenation (*n* = 4). (H) OGD/R‐induced changes in the viability of neuronal cells, treated with Annexin V and siRNA‐Tmem30a, measured using the CCK‐8 assay (*n* = 4). (I) OGD/R‐induced changes in the viability of neuronal cells, treated with anti‐PS antibody and siRNA‐Tmem30a, measured using the CCK‐8 assay (*n* = 4). (J) Representative images and quantification of CC3^+^ cells in primary neuron undergoing 2h OGD and 24h reoxygenation (*n* = 4). (K) Representative images and quantification of pMLKL^+^ cells in primary neuron undergoing 2h OGD and 24h reoxygenation (*n* = 4) Bar=10µm. **p < 0.01, by one‐way ANOVA with Bonferroni's multiple comparison in (B), (F), (G), (H), (I), (J), and (K).

To study the mechanisms underlying the association among *Tmem30a* levels, PS exposure, and Annexin V changes, first we explore the association between *Tmem30a* levels and various duration of ischemia in a permanent oxygen glucose deprivation (OGD) model (Figure 4A). We found that *Tmem30a* mRNA levels were increased initially (2 h), but continuously decreased with the extended duration (4 and 6 h) of ischemia (Figure 4B). Then, in a 2h OGD and reoxygenation (OGD/R) model, increased *Tmem30a* mRNA levels were observed when we collected neuron at 0, 2, 3, 6, and 24 h after reoxygenation, the highest *Tmem30a* level at 3 h (Figure S5A, B).

Then, we explored the association between Tmem30a level and PS exposure. When testing for PS exposure, Annexin V‐FITC staining is frequently utilized. Early apoptosis is indicated by Annexin V‐FITC (+) and PI (−) staining, whereas late apoptosis is represented by dual positive staining of Annexin V and PI [29, 30]. Two‐hour OGD resulted in significantly increased Annexin V+/PI− neurons and Annexin V+/PI+ neurons, 4h permanent OGD exhibited significantly decreased Annexin V+/PI− neurons but parallel Annexin V+/PI+ neurons, and no Annexin V+/PI− or Annexin V+/PI+ neurons after the 6‐h permanent OGD (Figure 4C). These data suggest that permanent ischemia led to a decreased *Tmem30a* level and a shift from early neuron cell death to late death. Next, we performed OGD/R studies to further explore the association of *Tmem30a* and PS exposure (Figure 4D). siRNA‐*Tmem30a* administration reduced *Tmem30a* expression and increased PS exposure in neurons after 2h OGD and 3h reoxygenation (Figure 4E, F). These findings indicated that reducing *Tmem30a* level after OGD/R led to a higher PS exposure.

Next, we used two PS blockers, Annexin V recombinant protein and anti‐PS antibody, to explore the association between PS blocking and cell viability in OGD/R models. Recombinant Annexin V did not induce pSIVA^+^ (PS exposure) in normal neuron culture, but reduced pSIVA^+^ neuron after OGD/R (Figure 4G). Moreover, both PS blockers increased cell viability after OGD/R (Figure 4H, I). More importantly, we found that siRNA *Tmem30a* could offset these protective effects by PS blockers based on an OGD/R cell model (Figure 4H, I). These findings indicated that *Tmem30a* was necessary for neural protection due to less PS exposure in OGD/R models.

Furthermore, we explored potential mechanisms. We found that CC3^+^ MAP2 cells (Figure 4J), pMLKL^+^ MAP2 (Figure 4K), and pRIPK1^+^ MAP2 (Figure S5C) were increased after OGD/R, while CC3^+^ MAP2, pMLKL^+^ MAP2, and pRIPK1^+^ MAP2 neurons were reduced by administrating recombinant Annexin V. Moreover, recombinant Annexin V treatment did not increase CC3^+^ NeuN, pMLKL^+^ NeuN, and pRIPK1^+^ NeuN under non‐OGD condition. These data indicated that recombinant Annexin V decreased apoptosis and necroptosis of neurons after OGD/R.

### 2.5 Upregulation of *Tmem30a* Confers Neurological Benefits in Ischemia/Reperfusion Models

**FIGURE 5:**
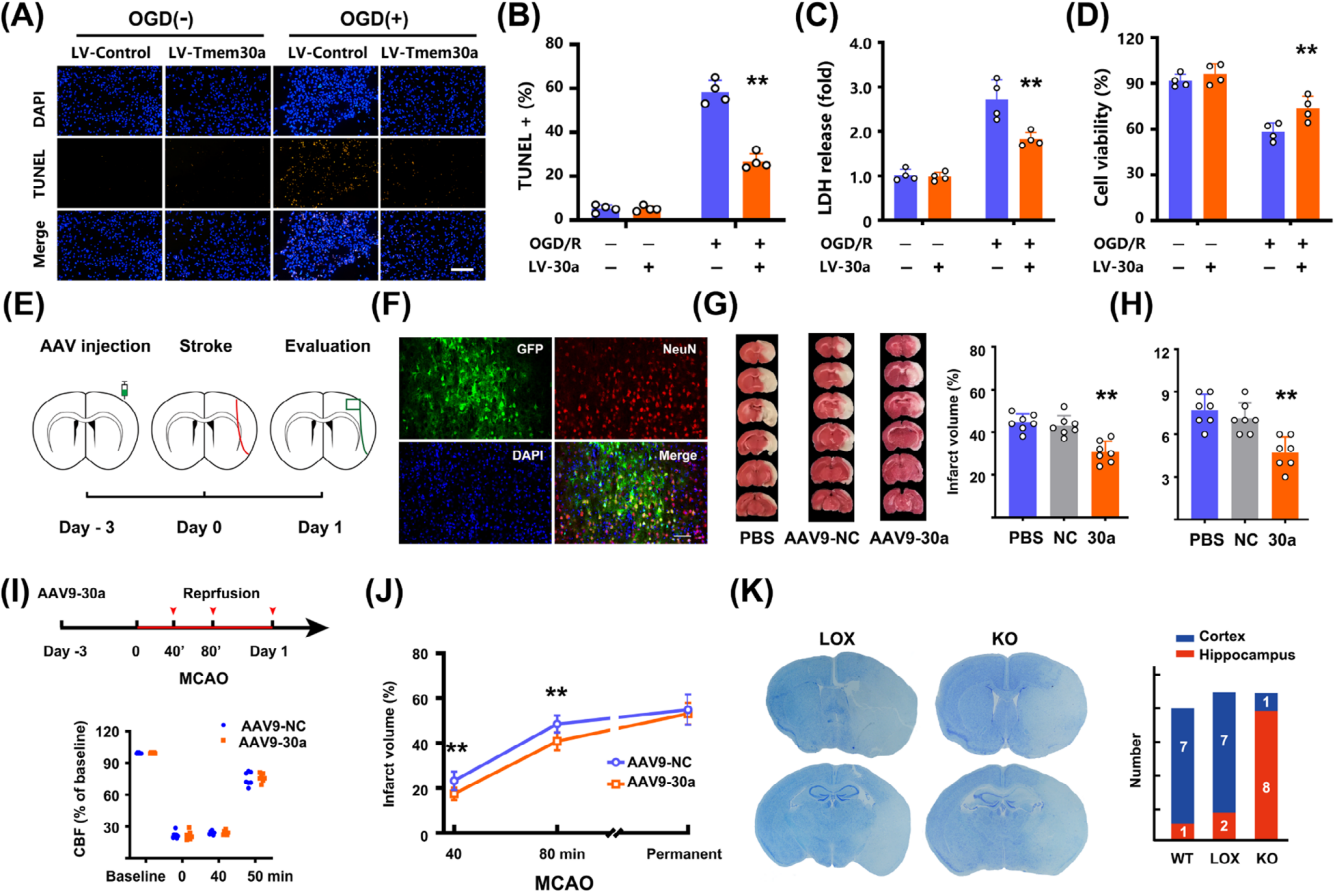
Upregulating Tmem30a mitigates ischemic brain injury. (A) Terminal deoxynucleotidyl transferase dUTP nick end labeling (TUNEL) staining in primary neurons transfected with lentivirus‐Tmem30a or lentivirus‐NC after a 2 h OGD and 24 h reoxygenation. Bar = 100 µm. (B) Percentages of TUNEL positive cells in primary neurons after OGD/R (*n* = 4). (C) LDH (lactate dehydrogenase) release after OGD/R. (D) Analysis of cell viability after OGD/R. **p < 0.01, by one‐way ANOVA with Bonferroni's multiple comparison in (B), (C), and (D). (E) Schematic of AAV9‐Tmem30a or AAV9‐control pre‐injection in rat tMCAO (transient MCA occlusion) models. (F) Viral GFP expression (green, AVV9− Tmem30a) co‐localized with NeuN (red). Bar = 100 µm. (G) Representative images and quantification of TTC staining in brain sections 24 h after 60‐min tMCAO (*n* = 7). (H) Neurological assessments in rat tMCAO models (transient MCA occlusion) receiving AAV9‐Tmem30a or AAV9‐NC after stroke. (I) Schematic of AAV9‐Tmem30a injection and different MCAO durations in mice and LSI recordings for CBF levels compared to baseline over ischemia and reperfusion. (J) Occlusion time‐dependent changes in infarct volumes in mice receiving AAV9‐Tmem30a compared to those with AAV9‐NC when undergoing transient and permanent MCAO 24 h after stroke (*n* = 7). **p < 0.01, two‐way ANOVA. (K) Representative Nissl images of ipsilateral brain regions in Tmem30aloxP/loxP and Tmem30a FnKO mice 24 h after 60min tMCAO (transient MCA occlusion). Numbers of models exhibiting hippocampus damages (in red) 24 h after stroke.

To explore the role of *Tmem30a*, we over‐expressed *Tmem30a* in neurons and subjected them to 2‐h OGD/R as an in vitro ischemia/reperfusion model. We observed that TUNEL+ percentage was significantly decreased in LV‐Tmem30a neurons after OGD, as compared with LV‐negative control (NC) neurons (Figure 5A, B). We also found that cell viability was markedly increased in LV‐Tmem30a‐treated neurons after OGD/R (Figure 5C, D). Collectively, these data suggested that upregulation of *Tmem30a* alleviated OGD/R‐induced neuron death.

We further explored whether overexpressing *Tmem30a* in vivo also reduced ischemic damages. Rats were injected with AAV9‐Tmem30a or AAV9‐NC into MCA‐supplying regions (Figure 5E). Viral GFP (green), anchored with *Tmem30a*, mainly co‐localized with NeuN (Figure 5F). Additionally, AAV9‐Tmem30a injection nearly doubled the expression of *Tmem30a* (Figure S6A) and AAV9‐Tmem30a was more notable when compared with the counterpart side (Figure S6B). In Figure 5G, overexpressing *Tmem30a* reduced cerebral infarct volumes when compared to models receiving vehicle and AAV9‐NC 24 h after stroke. In addition, overexpressing *Tmem30a* also significantly decreased both 12‐point (Figure 5H) and longa scores (Figure S6C), as compared to those receiving vehicle and AAV9‐NC. Finally, overexpression of *Tmem30a* also reduced apoptosis marker cleaved caspase‐3 (CC3) and necroptosis marker (p‐MLKL) and increased anti‐apoptosis marker Bcl‐xL and BCL‐2 in the penumbra regions 24 h after stroke (Figure S6D–I). These data indicated that decreased apoptosis and necroptosis in the penumbra might account for these neurological benefits.

It is difficult to monitor real‐time cerebral blood flow (CBF) changes in rat models. Thus, we used a mouse model of MCAO and monitored CBF changes to rule out the potential interference of perfusion status. AAV9‐Tmem30a were injected to the prospective stroke sites 3 days before stroke in C57BL/6 mice (Figure 5I). RT‐PCR results confirmed *Tmem30a* levels were increased (Figure S7A). We first developed a less severe model with 40min MCAO and 24‐h reperfusion. CBF levels were comparable between two groups (Figure 5I). AAV9‐Tmem30a injection led to a smaller infarct size and a better neurological score than those with AAV9‐NC (Figure 5J, Figure S7B). We also repeated these experiments with a more severe 80‐min MCAO and 24‐h reperfusion. Similar results were also observed (Figure S7C–E). However, there were no differences in both infarct sizes (Figure 5J) and neurological score when using a permanent occlusion model in mice (Figure S7F–H). The above data indicated that *Tmem30a* upregulation could mitigate penumbra damages after ischemia/reperfusion, but not stroke damages after permanent ischemia.

To further confirm the role of *Tmem30a*, we generated neuron‐specific *Tmem30a*‐deficient mice. However, these mice, induced by Nestin‐Cre, were not viable [17]. Thus, we generated a *Tmem30a* forebrain‐neuron‐specific knockout mouse (designated *Tmem30a* FnKO mice) by crossing *Tmem30a* ^loxP/+^; Cam2‐Cre mice to *Tmem30a* ^loxP/loxP^ animals. The *Tmem30a* ^loxP/loxP^ mice were used as controls. *Tmem30a* FnKO mice showed no change in the MCA area (Figure S8A, B) or alteration of CBF before, during, and after 60 min MCAO (Figure S8C, D) compared to *Tmem30a* ^loxP/loxP^ littermate mice, indicating that the cerebrovascular system was not affected by *Tmem30a* deficiency. Moreover, *Tmem30a* FnKO mice was comparable to wild‐type (WT) C57BL/6 mice in the Nissl and HE staining of hippocampus and neocortex.

Then, we explored the effect of focal ischemia on *Tmem30a* forebrain‐specific knockouts. Previously, the striatum and hippocampus were considered as a representative core area and penumbra area, respectively, in rodent MCAO models [31].

In this study, eight out of nine *Tmem30a* FnKO mice exhibited a severe loss of hippocampal neurons, as compared with only one out of eight WT littermates and two out of nine *Tmem30a* ^loxP/loxP^ mice after 60min MCAO and 24h reperfusion (Figure 5K). Moreover, *Tmem30a* FnKO mice also exhibited a larger infarct size in TTC staining after MCAO, as compared with their WT littermates and *Tmem30a* ^loxP/loxP^ mice (Figure S8E, F). Therefore, compared to their WT littermates and *Tmem30*a^loxP/loxP^ mice, *Tmem30a* FnKO mice were more likely to have ischemia injury in forebrain areas.

Taken together, these multiple independent lines of evidence suggest that upregulation of *Tmem30a* will confer neurological benefits after stroke, but neurological benefits are notable in models of ischemia/reperfusion, not in those of permanent ischemia.

### 2.6 *Tmem30a* is Imperative for Neurological Benefits of Recombinant Annexin V

Both recombinant Annexin V and upregulating Tmem30a led to neurological benefits in ischemia/reperfusion models. Then, we further explored whether *Tmem30a* is necessary for penumbra protection due to Annexin V administration (PS blocking) after stroke.

**FIGURE 6:**
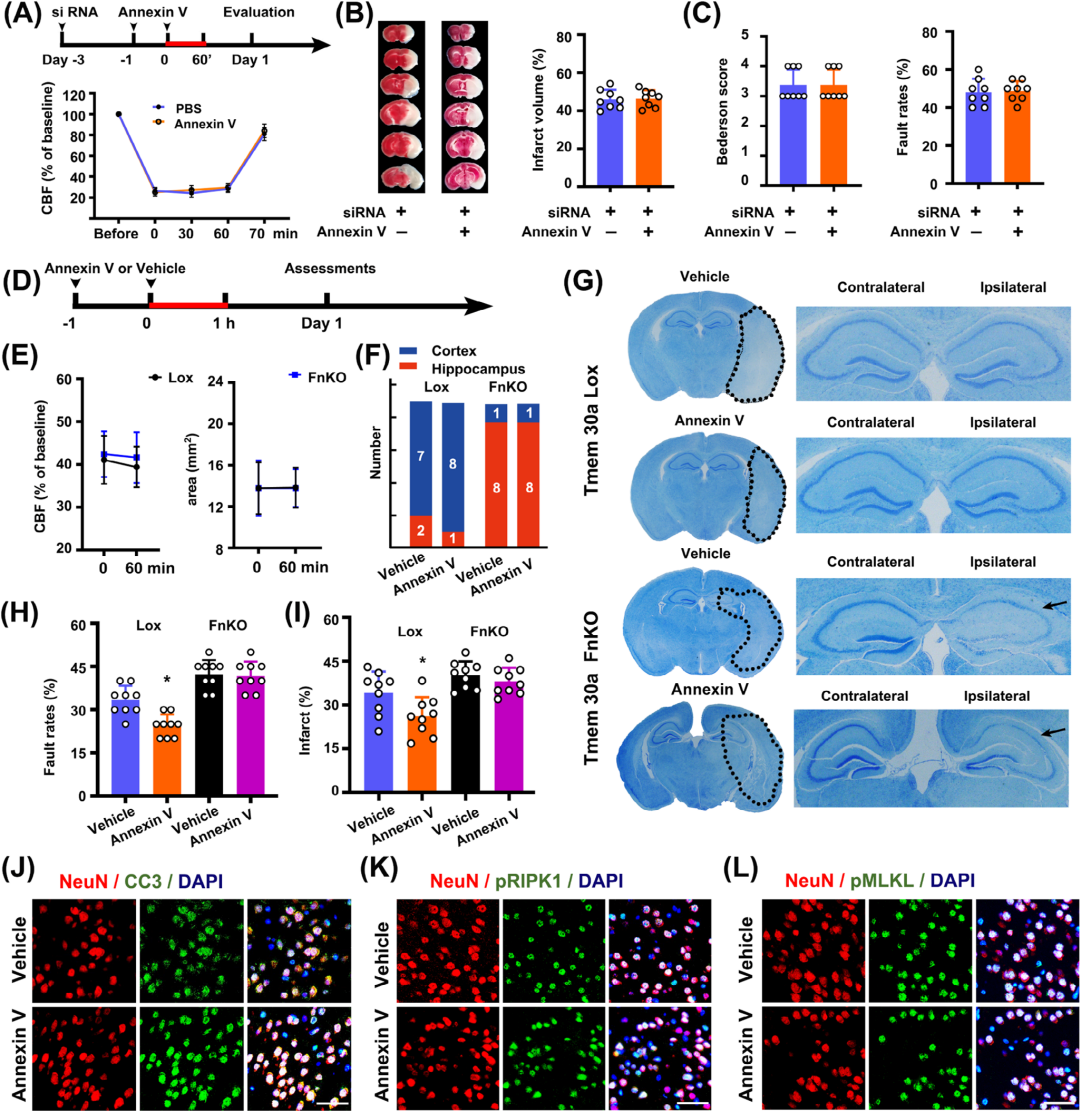
Tmem30a is imperative for neuroprotection of PS blocking after stroke. (A) Schematic of intraventricular injection of siRNA‐Tmem30a or siRNA‐NC and intravenous administration of Annexin V. CBF levels were comparable between two groups (*n* = 6). (B) Representative images and quantification of stroke volumes 24 h receiving siRNA‐Tmem30a mouse tMCAO (transient MCA occlusion) models with or without Annexin V (*n* = 8). (C) Neurological scores 24 h after stroke receiving siRNA‐Tmem30a mouse tMCAO (transient MCA occlusion) models with or without Annexin V (*n* = 8). (D) Schematic of Annexin V treatment in Tmem30a^loxP/loxP^ mice and Tmem30a FnKO tMCAO (transient MCA occlusion) mice (*n* = 18). (E) Graphs showing CBF levels and penumbra areas during 60 min tMCAO (transient MCA occlusion). (F) Numbers of models exhibiting hippocampus damages (in red) 24 h after stroke. (G) Representative Nissl images of brain slice showing infarct (in dash line) and hippocampus damages (black arrow) 24 h in Tmem30a^loxP/loxP^ mice and Tmem30a FnKO tMCAO (transient MCA occlusion) mice. (H) Foot fault test results 24 h after stroke onset (*n* = 9). (I) Infarct sizes among different groups 24 h after stroke (*n* = 9). *p < 0.05, by one‐way ANOVA with Bonferroni's multiple comparison in (H) and (I). (J) Representative images of CC3^+^ neurons in penumbra regions 1 day after MCAO. Bar = 10 µm (*n* = 4). (K) Representative images of pRIPK1^+^ neurons in penumbra regions 24 h after tMCAO. Bar = 10 µm (*n* = 4). (L) Representative images of pMLKL^+^ neurons in penumbra regions 1 day after stroke. Bar = 10 µm (*n* = 4).

Intracerebroventricular injection of siRNA‐Tmem30a decreased *Tmem30a* mRNA levels in cortex, increased infarct volumes, and exacerbated neurological scores in mouse models subjected to 60 min ischemia/24 h reperfusion, as compared with those receiving siRNA‐NC (Figure 6A, Figure S9A–E). Then, we tested whether Annexin V administration could mitigate neurological damages in mice receiving siRNA‐Tmem30a. However, Annexin V treatment could not reduce infarct sizes and improve neurological functions after 60 min ischemia/24 h reperfusion in mice receiving siRNA‐Tmem30a pre‐injection (Figure 6B, C). Moreover, injection of Annexin V could not reduce the number of TUNEL^+^ cells after 60 min ischemia/24 h reperfusion in mice receiving siRNA‐Tmem30a pre‐injection (Figure S9F, G).

These data suggest that neurological benefits of Annexin V treatment might be dependent on Tmem30a expression.

Furthermore, we administrated Annexin V in both *Tmem30a* FnKO and *Tmem30a^loxP/loxP^* mice to find out whether *Tmem30a* is imperative for attenuating penumbra damages by reducing PS exposure after stroke. There were no significant differences in CBF levels and penumbra areas during the 60‐min MCAO between the two groups based on LSI recordings (Figure 6D, E). But, Annexin V administration did not reduce the damage in hippocampus regions in *Tmem30a* FnKO mice, which were notably different from *Tmem30a^loxP/loxP^* mice (Figure 6F, G). Additionally, Annexin V treatment only reduced infarct sizes and improved neurological scores 24 h after stroke in *Tmem30a^loxP/loxP^* mice, while it did not do so in *Tmem30a* FnKO mice (Figure 6H, I). These findings further proved prevention of PS exposure by Annexin V is dependent on *Tmem30a* for neuroprotection after stroke.

Lastly, we also examined apoptosis and necroptosis markers in penumbra areas 24 h after 60min ischemia. We found that CC3^+^, pMLKL^+^, and pRIPK1^+^ neurons were decreased after Annexin V administration in *Tmem30a^loxP/loxP^* mice, but there were no differences in CC3^+^, pMLKL^+^, and pRIPK1^+^ neurons in *Tmem30a* FnKO mice (Figure 6J–L, Figure S10A–C).

## 3 Discussion

Internal survival signaling cascades are activated to prevent cell death when the cell is under danger [3, 32]. The penumbra, a potentially viable but still hypo‐perfused brain area when ischemic stroke occurs, is an excellent in vivo model for studying the mechanisms and potential translation of these survival signaling signals [33, 34]. We found that plasma Annexin V levels and *Tmem30a* in the penumbra was increased, both of which reduced PS exposure, apoptosis, and necroptosis after stroke, consequently improving neurological outcomes in rodent models of stroke. Furthermore, recombinant Annexin V, with a high affinity to PS, exerts neuroprotective effects, dependent on existence of *Tmem30a*, in both in vivo and in vitro ischemia/reperfusion models, but not in a permanent model.

“Eat‐me” signals might be translational targets for ischemic stroke. Firstly, protection imparted by *Tmem30a* or PS blockage has been found in other similar but no stroke scenario in previous studies. As reported, blocking PS reduces LPS‐induced neuronal death [11]. *Tmem30a* was imperative for keeping PS inside and maintaining cell membrane in fetal liver erythropoiesis [14]. Moreover, low glutamate levels led to reversed PS exposure without neuronal death or loss in vitro, while high glutamate concentrations caused irreversible PS exposure and loss or death of neurons [35, 36]. These previous findings acted as the rationale for our hypothesis. Our main finding was that neuroprotective strategy of reducing PS exposure in the early stage was feasible in the context of clinically relevant in vivo and in vitro ischemia/reperfusion models.

Second, we also observed the increased *Tmem30a* was linked to less PS exposure and neurological benefits in models of ischemia/reperfusion, but not in permanent ischemia. These findings provide a prerequisite (restoring blood perfusion) for neuroprotection by the increased *Tmem30a* and the reduced PS exposure. Thus, PS mitigation strategy might have the potential for cyto‐protection in stroke, especially in the new time of highly reperfusion. These findings offered a fresh framework for enhancing the results of acute ischemic stroke [37, 38].

*Tmem30a* was imperative for the neuroprotection of Annexin V after stroke. Previous studies have found *Tmem30a* mRNA was expressed in similar amounts in various tissues and played important roles in these tissues [39]. Deficiency of *Tmem30a* led to serious consequences, such as intrahepatic cholestasis in liver‐specific knockout mice and severe anemia in hematopoietic‐specific knockout mice [14, 40]. However, these studies only addressed the biological functions of *Tmem30a*. Recently, a study highlighted the multifaceted role of *Tmem30a* in B‐cell lymphomagenesis, revealing that the vulnerabilities in lymphoma cells caused by *Tmem30a* expression can be therapeutically exploited [41]. In our study, we uncovered a critical role of *Tmem30a* in these stressed but still viable neurons and elucidated it as a potentially translational target in stroke treatments. Our findings not only found that *Tmem30a* was shortly upregulated in the penumbra after stroke, but proved the association of *Tmem30a* upregulation with better neurological outcomes. The upregulation of *Tmem30a* in neurons shortly after focal ischemia may be due to the self‐preservation mechanisms after stroke and may act as a “help‐me” signal in the acute stroke [42]. Additionally, we also observed that the PS‐neuroprotection induced by Annexin V binding was not present when *Tmem30a* was absent, either by knockout, siRNA inhibition, or longer ischemia. This may have also resulted from the crucial function of *Tmem30a* in moving PS into the inner layer of the cell membrane. When ischemic events were extended, expression of *Tmem30a* was significantly reduced. Thus, *Tmem30a* may also be a therapeutic target in the acute stage of stroke in the era of recanalization, helping to increase the opportunity of reperfusion therapy and adjunctive cytoprotective treatments [43].

Prior to this, Annexin V conjugates offered rapid and accurate detection techniques for researching PS externalization, a marker of the intermediate phases of apoptosis [44]. Moreover, Annexin V protein was reported to inhibit phagocytosis of apoptotic and necrotic cells not only by reducing PS exposure through its binding to the apoptotic cell surface, but also by influencing the progression of the apoptotic cell death program, such as delaying the activation of caspase 3 [45, 46]. More importantly, previous studies have shown that Annexin V can permeate through the BBB when injected intravenously, demonstrating its ability to have neuroprotective benefits [12, 47, 48]. Taken together with previous findings, our study provides evidence for a novel clinical potential of Annexin V in protecting the penumbra after stroke.

However, in both rodents and patients, the plasma concentration of Annexin V is low and stable under normal physiological conditions, and it modestly increases following a stroke. According to a prior investigation, a high concentration of Annexin V was necessary to inhibit phagocytosis [49].

There are several intriguing questions or limitations might exist in this study. First, our study suggests that Tmem30a in the central and Annexin V in the plasma protect penumbra, but whether a crosstalk exist and the underlying mechanisms remain unclear. How ischemia/reperfusion damages in the penumbra affects peripheral changes should be explored. Furthermore, we only used healthy young male models, tested translational neuroprotection in acute ischemic stroke, and highlighted the well‐known effects of Annexin V in reducing PS exposure. Future studies are needed to carefully delineate upstream mechanisms, not just the apoptosis and necroptosis in this study. Finally, we only proved Annexin V led to less PS exposure and increased cell viability in cell culture. Due to the existence of BBB and a relatively large molecular weight of Annexin V (about 36 kD), it is difficult to image PS exposure by using Annexin V connected with an imaging agent directly at present [50].

Taken together, our data suggested that PS exposure, a previously well‐known eat‐me signal, is a novel target for the penumbra protection after ischemic stroke. *Tmem30a* upregulation and an increased plasma level of Annexin V reduced PS exposure in the penumbra after stroke, consequently leading to a better neurologic outcome. Tmem30a is essential for reducing PS exposure in the penumbra after ischemic stroke.

## 4 Materials and Methods

### 4.1 Study Population

The human study part 1 was carried out at Xuanwu Hospital Capital Medical University, one center in China, in compliance with the Declaration of Helsinki. We recruited 22 patients who had experienced their first ischemic stroke; both informed consent and the study methodology were approved by the institutional review board; the inclusion criteria were as follows: age ≥ 18 years; the patient had to have experienced their first ischemic stroke and present within 12 h of the onset of symptoms, which was defined by the “last known.” Neurologists also recruited 22 age‐matched healthy controls from the patients' relatives, primarily spouses, to assess for cerebrovascular events.

The clinical human study part 2 was conducted at one center in the China (Xuanwu Hospital Capital Medical University) and performed in accordance with the principles of the Declaration of Helsinki. Both informed consent and the study methodology were approved by the institutional review board. Consecutive eligible patients, who suffered from a proximal arterial occlusion in the anterior circulation artery and were treated with endovascular treatment (EVT) within 6h after symptom onset, were enrolled.

The prerequisites for inclusion were as follows: 18 years of age or older; digital‐subtraction angiography, computed tomographic angiography, or magnetic resonance angiography–detected acute ischemic stroke caused by blockage of the distal intracranial carotid artery, M1 or M2 segment of the middle cerebral artery, or A1 or A2 segment of the anterior cerebral artery; evaluation of the ischemic penumbra prior to EVT was done using computed tomographic perfusion or magnetic resonance perfusion; femoral artery puncture (EVT) was performed within 6 h of the onset of symptoms. The time the patient was last seen well was used to determine the onset of stroke symptoms [51, 52].

### 4.2 Animal Models

All animal treatments were carried out strictly in compliance with the National Institutes of Health's Care and Use of Laboratory Animals guidelines, and all experimental methods were authorized by Capital Medical University's Institutional Animal Investigation Committee. A total of 175 (21–22 g) male C57BL/6 mice were purchased from SiPeiFu Biotechnology Co. (Beijing, China) and 50 knockout male mice (21–22 g) from C57/BL6 background were provided by Professor Xianjun Zhu. Vital River Laboratory supplied 27 male SD rats weighing between 280 and 300 g. They were kept in clear cages (five or less per cage), free access to food and water, and an ambient temperature of 21–25°C and 40%–80% humidity. Wild‐type SD rats were randomly assigned to sham (*n* = 10), tMCAO (*n* = 10), and AAV9‐30A (*n* = 7), while knockout mice were randomly divided into TMEM30A Lox (*n* = 18), TMEM30A knockout (*n* = 18), and TMEM30A knockout plus Annexin V treatment (*n* = 14) groups.

The survival rate of permanent MCAO mice and the 7‐day tMCAO models was approximately 50%. The survival rate of animal was 100% in other types of models.

Exclusion criteria were as follows: Penumbra areas were defined as CBF values approximately 30%–50% of the baseline values during the 60‐min period. We operationally defined the core areas as perfusion deficits areas (< 50%) minus the penumbra areas. Excluded were any animals whose baseline LDF values did not significantly decrease to less than 30% during MCAO.

### 4.3 RNA‐Sequencing Analysis for Penumbra Tissues of Rhesus Monkeys

4.3.1

We operationally define penumbra as the mismatch between perfusion‐weighted imaging (PWI) and diffusion‐weighted imaging (DWI) in focal ischemic model of Macaca mulatta. We then obtained the cerebral cortex, as shown by red dotted circle in Figure 1C, and extracted total RNA with TRIzol protocol (Invitrogen Canada, Burlington, ON, Canada). After analyzing the purity and integrity, 2 µg RNA of each sample was used for generating cDNA libraries using NEB Next Ultra RNA Library Prep Kit for Illumina (#E7530L, NEB, USA). Then, using the Illumina HiseqTM 2500 (Illumina, San Diego, CA, USA) and the manufacturer's instructions, RNA sequencing was carried out at Annoroad Gene Technology (Beijing, China). Pairwise readings of 150 bp were produced for further data analysis. Further detailed methods for RNA‐Sequencing analysis can be found in the Supplementary information.

### 4.4 Neurological Deficits

Longa score and 12‐scoring scale were used for the analysis of neurological deficits in rats. Bederson score and Foot fault were examined for evaluation of neurological deficits in mice [53].

### 4.5 Bederson Score for Mouse Models

The Bederson score was used to assess neurological deficits during the acute phase of stroke [53]. In short, animals that exhibit forelimb bending without any other anomaly are classified as having Grade 1 (minor deficiencies). Animals with forelimb flexion and reduced resistance to lateral push toward the paretic side are classified as having Grade 2 (modest defects). Animals that exhibit circling behavior in addition are given a Grade 3 rating (severe defects). Grade 4 animals are those that rotate longitudinally after a stroke, while Grade 5 animals do not move.

### 4.6 Cell Viability Assay

At 24 h following OGD, cell viability was assessed using CCK8 (DojinDo, Tokyo, Japan) by observers who were blind to the experimental groups. In short, each culture well received 10 µL of CCK8 reagent, and the neurons were then treated for 2 h at 37°C. The absorbance reader (Thermo Scientific, MA, USA) was used to measure the absorbance at 450 nm. The percentage of the naive control was used to represent the neurons' vitality.

### 4.7 ELISA

In brief, both plasma and brain sample isolated and homogenized mechanically according to instruction. All procedures were based on instruction for Annexin V ELISA testing. The levels of Annexin V in tissue supernatants and plasma samples were assessed using an enzyme‐linked immunosorbent assay Kit (Cloud‐Clone Corp, Wuhan, China). Using a Varioskan LUX multi‐function microplate reader (Thermo Scientific, MA, USA), the absorbance at 450 nm was determined. The standard curve of Annexin V was used to measure the samples' concentration.

### 4.8 Annexin V Treatment

Annexin V was purchased from Biovision and dissolved in PBS. Annexin V (1 mg/kg) or PBS was intravenously injected into wild‐type mice, *Tmem30a^loxP/loxP^ Tmem30a* HnKO, once the day prior to MCAO onset. Another injection of Annexin V was given right before MCAO. To assess the putative therapeutic potential of Annexin V in stroke outcomes, we assessed the effect of PS blocker at different time points (0, 30, 60, and 90 min) after MCAO.

### 4.9 Statistical Analysis

We performed statistical analysis using SPSS 19.0. All data are shown as the mean ± SEM and were analyzed using either two‐way ANOVA, one‐way ANOVA followed by Bonferroni's post hoc test (for comparisons of more than two groups), or two‐tailed Student's *t* test (for comparisons of two groups). At *p* < 0.05, differences were deemed significant.

## Author Contributions

C.W. made substantial contributions to the acquisition and analysis of clinical biomarker data and assisted with the experiment. Y.D. and J.G. carried out the experiment, assisted with the experimental design and data analysis. D.W., Y.D. and J.G. wrote the manuscript. J.H. and S.X. helped with immunofluorescence staining and data analysis. J.H. performed animal experiments. C.Z. and Y.D. provided technical help in RNA‐seq analysis and revised the manuscript. S.X. and J.H. assisted with animal experiments. X.Z. provided Tmem30a KO mice and the Tmem30aloxP/loxP. G.L. and Y.D. assisted with the experimental design and revised the manuscript. D.W. and X.J. and X.Z. conceived the project, supervised all experiments, and wrote and revised the manuscript. All authors fulfill the criteria for authorship. All authors have read and approved the final manuscript.

## Ethics Statement

This patients study was authorized by the institutional review board of Xuanwu Hospital Capital Medical University (No. 2017‐030), and was carried out in accordance with the Declaration of Helsinki's tenets. Prior to enrollment, written informed permission was obtained from each patient or their legally permitted representative. The Capital Medical University Institutional Animal Care and Use Committee accepted the rhesus monkey trials in accordance with protocols (No.: AEEI‐2021‐064). Additionally, every experiment adhered to the Guide for the Care and Use of Laboratory Animals and national regulations. The Institutional Animal Care and Use Committee of Xuanwu Hospital, Capital Medical University, gave its approval to the rodent research (No.:XW‐20220905‐1, No.:XW‐20220620‐1).

## Conflicts of Interest

The authors declare no conflicts of interest.

## Supporting information



Supporting Information

## Data Availability

Data from cardioembolic stroke samples and normal control samples has been deposited in GEO: GSE58294. The data that support the findings of this study are available from the corresponding author upon reasonable request. Tmem30a^loxP/loxP^ mice are available from the corresponding author (Di Wu) upon request.
