# Supplementary material for: Mitigating Early Phosphatidylserine Exposure in a Tmem30a‐Dependent Way Ameliorates Neuronal Damages After Ischemic Stroke
Source: MedComm (2020). 2025 Mar 18;6(4):e70140. doi: 10.1002/mco2.70140 (PMC11914776; doi:10.1002/mco2.70140)
Supplement: Supplementary file 1 — Supporting Information [file MCO2-6-e70140-s001.docx]

**Title Page**

**A short informative title**

Mitigating early phosphatidylserine exposure in a Tmem30a dependent way ameliorates neuronal damages after ischemic stroke

**A short running title**

Mitigating PS in Tmem30a dependent way in stroke

**Authors**

Chuanjie Wu^1,#^, Jiaqi Guo^1, #^, Yunxia Duan^1,2,3, #^, Jiachen He^1^, Shuaili Xu^1^, Guiyou Liu^3^, Chen Zhou^3^, Yuchuan Ding^4^, Xianjun Zhu^5*^, Xunming Ji^1,2,3*^, Di Wu^1,2,3*^

^1^Department of Neurology and China-America Institute of Neuroscience, Beijing Institute of Geriatrics, Xuanwu Hospital, Capital Medical University, Beijing 100053, China

^2^Beijing Key Laboratory of Hypoxia Conditioning Translational Medicine, Beijing 100053, China

^3^Center of Stroke, Beijing Institute for Brain Disorders, Capital Medical University, Room 713, Morphology Building, No.10, Xitoutiao, You’an Men Wai, Fengtai District, Beijing 100069, China

^4^Department of Neurosurgery, Wayne State University School of Medicine, Detroit, MI, USA

^5^The Sichuan Provincial Key Laboratory for Human Disease Gene Study and Department of Laboratory Medicine, Center for Medical Genetics, Sichuan Provincial People's Hospital, University of Electronic Science and Technology of China, Chengdu, Sichuan 610072, China

^#^ These authors contributed equally to this work.

^*^**Corresponding author**

Di Wu, PhD,

Department of Neurology and China-America Institute of Neuroscience,

Xuanwu Hospital,

Capital Medical University,

Beijing 100053, China

Email: [seadi-wu@163.com](mailto:seadi-wu@163.com)

Or

Xunming Ji, MD, PhD,

Department of Neurology and China-America Institute of Neuroscience,

Xuanwu Hospital,

Capital Medical University,

Beijing 100053, China

Email:[jixm@ccmu.edu.cn](mailto:jixm@ccmu.edu.cn)

Or

Xianjun Zhu, PhD,

The Sichuan Provincial Key Laboratory for Human Disease Gene Study and Department of Laboratory Medicine,

Center for Medical Genetics, Sichuan Provincial People's Hospital,

University of Electronic Science and Technology of China,

Chengdu, Sichuan 610072, China

Email: [xjzhu2@126.com](mailto:xjzhu2@126.com)

**Supplemental Methods**

**Human study part 1**

As soon as the eligible patients were admitted, a venous blood sample was taken. Before being analyzed, whole blood samples were divided into vials, centrifuged right away into plasma, and kept in a refrigerated at -80°C. ELISA was used to measure the patient's plasma levels of Annexin V in accordance with operating instructions. Board-certified laboratory workers who were not privy to the clinical data conducted the analyses using the same batch of reagents. 1.6% to 6.8% were the intra-assay coefficients of variance, and 2.2% to 8.1% were the inter-assay coefficients of variation.

**Human study part 2**

Exclusion criteria included: pharmacological sedation and intubated patients if a neurologist or emergency physician did not obtain a baseline National Institutes of Health Stroke Scale (NIHSS) score prior to sedation or intubation; seizures at the onset of the stroke that would preclude obtaining a baseline NIHSS; having known hemorrhagic diathesis, coagulation factor insufficiency, or oral anticoagulant therapy with INR＞3; and being treated with alteplase more than 4.5 h after the onset of symptoms.

The NIHSS was used to measure the clinical stroke severity (range: 0-42, with higher values indicating more severe impairment) (22, 23). Early neurological improvement (ENI) was defined as a reduction of 8 points or more on the NIHSS or an NIHSS score of 0 or 1 at 24 h. The difference between NIHSS at baseline and at 24 h (ΔNIHSS 6h-24h) was used to assess the early clinical improvement.

A venous blood sample of eligible patients was collected as soon as possible after admission. The storage and detection methods of Annexin V levels were conducted as above. Before being analyzed, whole blood samples were divided into vials, centrifuged right away into plasma, and kept in a refrigerated at -80°C. ELISA was used to measure the patient's plasma levels of Annexin V in accordance with operating instructions. The analyses were performed with the same batch of reagents by board-certified laboratory technicians who had no access to the clinical data. The intra-assay coefficients of variation were 1.6% to 6.8%, and the inter-assay coefficients of variation were 2.2% to 8.1%.

All eligible patients (n=60) were screened using the above-mentioned criteria. Plasma Annexin V levels were compared between patients with ENI or without ENI. Following that, the patients were split into two groups: those who had penumbra and those who did not. In patients with and without penumbra, the relationship between Annexin V concentrations and △NIHSS was evaluated. The volume of penumbra was computed using automated software (e-Stroke, Neusoft, China). The volume of hypo-perfused tissue (time to maximal delay >6 s) minus the volume of ischemic core (cerebral blood flow<30% of that) in contralateral normal tissue was used to determine the volume of the penumbra. Patients with penumbra are characterized by an absolute volume of 15 mL or more for potential reversible ischemia, an infarct volume (ischemic core) of less than 70 mL, and a ratio of ischemic tissue volume to initial infarct volume of 1.8 or greater (54).

a ratio of ischemic tissue volume to initial infarct volume of 1.8 or higher, and an infarct volume (ischemic core) of less than 70 mL.

**Rats**

In this study, adult male Sprague-Dawley (SD) rats weighing between 280g and 300g (SPF grade; Vital River Laboratory Animal Technology Co.) were used. Each rat had unrestricted access to food and water. The temperature in the rising room ranged from 23°C to 25°C, and the relative humidity was between 40% and 80%.

**Mice**

It is difficult to monitor real-time cerebral blood flow (CBF) changes in rat models. Thus, we used a mouse model of the MCAO and monitored CBF changes to rule out the potential interference of perfusion status on stroke outcomes. C57BL/6 mice were purchased from SiPeiFu Biotechnology Co. (Beijing, China) hippocampus neuron specific KO *Tmem30a* KO mice and the *Tmem30a* loxP/loxP mice were provided by Professor Xianjun Zhu.

*Tmem30a^loxp/loxp^* mice were mated to CamII-Cre mice (B6. Cg-Tg (Camk2a-cre) T29-1Stl/J) to generated *Tmem30a^loxp/+^; CamII-C*re offsprings (55). *Tmem30a^loxp/+^; CamII-C*re were mated to *Tmem30a^loxp/loxp^* animals to generate *Tmem30a^loxp/loxp^* *CamII-C*re (named *Tmem30a* HnKO) mice.

Using Tmem30a gene primers and PCR method to amplify mouse tail genome DNA(*Tmem30a*-Loxp2-F, 5′-attccccttcaagatagctac-3 and *Tmem30a*-Loxp2-R, 5-aatgatcaactgtaattcccc -3). PCR Amplification was performed using a master mix (Invitrogen, USA). The first cycle consisted of 95 °C for 2 min, followed by 33 cycles of 94 °C for 15s, 58 °C for 20s and 72 °C for 30s. Cre was genotyped using generic Cre primers: Cre-F, TGCCACGACCAAGTGACAGCAATG, and Cre-R, ACCAGAGACGCAAATCCATCGCTC).

All animal experiments were conducted in accordance with the guidelines of the Animal Ethics Committee of Capital Medical University.

**Rhesus monkeys**

In order to reduce the biological gap between mouse and human, we used rhesus monkeys to explore potential changes in the brain in this study. Three mature male Macaca mulatta rhesus monkeys, ages 8-10, weighing 8.4-10.2 kg, were used in this study. Bacteria, ectoparasites, tuberculosis, Entamaebahistolytica, and B viruses were not found in any of all examined animals. In the same room, each monkey was kept in a different stainless steel cage. The primate subjects received twice-daily provision of standardized simian chow supplemented with fresh fruit, with continuous access to hydration. All experimental protocols were conducted with formal approval by the Institute of Lab Animal Sciences' Animal Use and Care Board at Capital Medical University. The Guide for the Care and Use of Laboratory Animals and national requirements were also followed in all investigations.

**Primary culture of cortical neurons**

We cultivated primary neurons as described in the earlier article (56). The cerebral cortex of 18-day-old SD rat embryos was used to harvest primary neurons, which were then supplied with 1% B27 (Grand Island, USA), 1% glutamate (1% Life Technology), and 1% penicillin/streptomycin (Life Technology).

The cells are maintained at 37°C in a humidified atmosphere with 95% O_2_ and 5% CO_2_. After one day of cultivation, neurons are switched to Neurobasal Medium and used after five more days.

**Case-control gene expression analysis**

We evaluated the potential differential mRNA expression of ANXA5 in 36 cardioembolic stroke samples and 23 normal control samples using a case-control gene expression datasets from GEO (GSE58294). <https://www.ncbi.nlm.nih.gov/geo/query/acc.cgi?acc=GSE58294>. Here, we used the online analysis program GEO2R to determine which genes were differentially expressed. (https://www.ncbi.nlm.nih.gov/geo/geo2r/) (Table S4) (20).

**Ischemia/Reperfusion models**

**Mice ischemic models**

Adult male mice were insensitive to tail pinch test after anesthesia with 70:30% N_2_O/O_2_, and maintained anesthesia with a nasal cone breathing 1.5% isoflurane. Using a nasal cone to inhale 1.5% isoflurane, the anesthesia was maintained. From the external carotid artery into the internal carotid artery, a 7-0 (in mice) monofilament with a silicon-coated tip (Doccol) was advanced to the MCA branching site in order to cause temporary localized ischemia. Adequate ischemia was verified using Continuous Laser Speckle Contrast Imaging (RWD Life Science Co., Shenzhen, China). Animals that did not exhibit a substantial decline in baseline LDF values to less than 30% during MCAO were excluded. CBF values between 30% and 50% of the baseline values were considered penumbra zones during the 60-min period (57,58). The core areas were operationally defined as locations with perfusion deficits (<50%) less the penumbra areas. The MCA of mice was blocked for 60 min, and then the monofilament suture was carefully removed to allow blood to flow again. A heating device with feedback control was used to keep the rectal temperature at 37°C .

**Rat ischemia model**

Adult male SD rats weighing between 280g and 320g were uesd. Rats were kept on 1.5% to 3% isoflurane while wearing a facemask after being sedated with 5% isoflurane and 70% N_2_O/O_2_ until the tail pinch test proved ineffective. We used a similar method to produce 2-h MCAO as we previously reported (59, 60). To induce transitory localized ischemia, a 4-0 (in rats) monofilament with a Doccol was inserted from the external carotid artery into the internal carotid artery and advanced to the MCA branching point. Rats were exposed to the MCA for 120 min.

**Monkey ischemic models**

In summary, as we previously described, a SilverSpeed-10 Hydrophilic micro-wire-equipped Prowler-10 micro-catheter (Codman) was inserted into the guiding catheter and guided to the distal end of the M1 segment of the right MCA in NHPs models (24). The clot was then moved into a micro-catheter, and two milliliters of saline were used to flush it into the end of the M1 segment. t-PA was administered via the microcatheter at a dose of 1.1 mg/kg. Prior to and 10 min following t-PA thrombolysis, cerebral angiography was conducted. A Magnetom Trio MRI Scanner was used for the MRI scan. In our previous study, we published MRI sequences and parameters (24).

**Laser speckle imaging**

Focal cerebral ischemia induction was achieved via filament-induced middle cerebral artery occlusion under general anesthesia. Surgical stabilization was ensured through stereotactic cranial fixation, effectively mitigating respiratory-associated motion artifacts during the procedure. Disinfect the skin at the top of the skull and make a longitudinal skin incision to expose the skull. Aim the direct center of the laser field at the anterior bregma of the skull, so that both sides of the skull are more completely presented in the laser field of view. After adjusting the distance between the laser probe and the skull, click to record the cerebral blood flow for about 10-15s to complete the recording of cerebral blood flow in this stage, and mark the name of this stage of recording on the software. 4 time points were selected for recording before the surgery, during occlusion onset, 60 min after occlusion, and 10 min after thrombectomy. The recording position of the same animal should be consistent as far as possible each time.

After the recording was completed, the left middle cerebral artery blood supply area was selected as region of interest 1 in the software, and the background was selected as region of interest 2 in order to eliminate the differences recorded at different times, and the cerebral blood flow laser speckle imaging degree of the two regions of interest in different stages was calculated respectively. The difference between area of interest 1 and area of interest 2 is the corrected blood flow degree in the left MCA supply area. The percentage of blood flow reduction after ischemia model making and the percentage of blood flow recovery after reperfusion were calculated using the baseline reading before surgery as standard 1.

In order to conduct in-depth analysis of cerebral blood flow changes, images at different time points after ischemia and reperfusion were selected to compare the area of the infarction core area and penumbra area (57). Excluded were any animals whose baseline LDF values did not significantly decrease to less than 30% during MCAO.

Penumbra areas were defined as CBF values approximately 30%-50% of the baseline values during the 60-min period (57, 58). We operationally defined the core areas as perfusion deficits areas (<50%) minus the penumbra areas. The was carefully removed. After blocking the mice MCA for 60 min, removing monofilament suture to restore blood flow. A heating device that was controlled by feedback was used to keep the rectal temperature at 37°C.

**Infarct size and neurological deficits**

Evaluate the infarct volume 24 h after reperfusion. After cutting six successive coronal brain slices, each measuring 2 mm in thickness for rats and 1 mm for mice, the slices were exposed to 2% TTC for 15 min at 37°C. Then take photos of all slices and calculate the infarct volume using Image J. The ipsilateral and contralateral hemispheres' non-infarcted regions, with the infarct region shown as a proportion of the latter.

**Foot-fault test**

After an ischemic stroke, The sensorimotor function of mice was assessed using the foot-fault test, also called the grid-walking task (61). The grid-walking apparatus, which had a rid area of 40 cm/20 cm/50 cm (length/width/height) and a mesh size of 12 mm, was produced with just minimal modifications as previously described (62). 24h following MCAO surgery, each mouse was set up separately on a wire grid and given 5min to wander about freely. If the animal reclined with the grid at wrist level or if the limb fell into the grid hole, a foot defect was noted. Researchers who were blind to the experiment counted the overall number of footsteps and foot defects. The formula foot faults/ (foot fault + non-foot fault steps) ×100% was used to get the ratio of foot faults.

**Stereotaxic injection of siRNA in mice and rats**

The powder of siRNA was synthesized by Ribobio (Guangdong, China). The siRNA sequence for rats (F’-GATGATTTACACCCAACACTACCAG；R‘-ATTCTTTCCTCCCATCCATGA), and for mice (F’-GAAGACAAACCGATTGCTCCA; R’-TGGCAACCAGAAACAACTCTAAC).

The powder of siRNA-negative control or siRNA-*Tmem30a* was dissolved with sterile RAase-free PBS. siRNA-negative control (siRNA-NC) or siRNA-*Tmem30a* were intracerebroventricularly injected into the rat or mouse brain 3 days before the MCAO procedure. In summary, Bregma was selected as the coordinate axis origin, and a hand-held cranium drill (RWD Life Science Co., Shenzhen, China) was used to drill a tiny hole at the stereotaxic coordinates.

After being given anesthesia, rats were attached to a stereotaxic device (Stoelting, Kiel, WI). After carefully mixing 3µL of siRNA-NC or siRNA-Tmem30a (1000 μmol/L), the rats were given an injection into the right cerebral ventricle (from bregma: posterior, −1.0 mm; lateral, −1.5 mm; ventral, −4.0 mm). The injection was then allowed to sit at room temperature for 20 min. The establishment of MCAO operation occurred three days after injection.

After being given anesthesia, C57BL/6 mice were attached to a stereotaxic device (Stoelting, Kiel, WI). After carefully mixing 1 µL of siRNA-NC or siRNA-Tmem30a (500 μmol/L), the mice were given an injection into the right cerebral ventricle (from bregma: posterior, −0.7 mm; lateral, −1 mm; ventral, −2.5 mm). The mice were then allowed to sit at room temperature for 20 min. The establishment of MCAO operation occurred three days after injection.

**Overexpression of *Tmem30a* in vitro**

**Virus vector GV492-*Tmem30a* production**

Shanghai GeneChem Company supplied the adeno-associated virus vectors used in this investigation. In short, pHelper and AAV9-GV388 plasmids were co-transfected into AAV-293 cells in addition to AAV9-Tmem30a (for both rat and mouse Tmem30a gene: NM_001004248-T2A). Following centrifugation, splitting, and ultra-filtration, the AAV-293 cell supernatant was concentrated and purified. qRT-PCR was used to measure the virus titer (1.07E+13 v.g./ml). As a negative control, the AAV9-empty vector was employed. To confirm that the vectors successfully transfected the intended area of the brain, AAV9 encoding the gene for green fluorescent protein (AAV9-GFP) was utilized.

**Stereotaxic injection of AAV9-Tmem30a in mice and rats**

A total of 1×10^13^ genome copies of AAV9-Tmem30A and AAV-NC were injected into the rat brain in the peri-ischemic cortex 3 days before the MCAO procedure. Briefly, choose bregma as the origin of the coordinate axis and drill a small hole in the stereotactic coordinates with a hand-held skull drill (RWD Life Sciences Co., Ltd., Shenzhen, China).

After being given anesthesia, rats were attached to a stereotaxic device (Stoelting, Kiel, WI). Rats' right cerebral cortex (from the bregma: posterior, −2.0 mm; lateral, −2.0 mm; ventral, −2.5 mm) received 3 µL of AAV9-NC or AAV9-Tmem30a at a rate of 0.3 µL/min for 10 min. To guarantee that the virous was completely dispersed, the needle was left in place for 5min before being removed. The establishment of MCAO operation occurred three days after injection.

After being given anesthesia, C57 mice were attached to a stereotaxic device (Stoelting, Kiel, WI). Mice were given injections of 1 µL of either AAV9-NC or AAV9-*Tmem30a* at a rate of 0.3 µL /min for 4 min into the right cerebral cortex (from bregma: posterior, −1.0 mm; lateral, −1.0 mm; ventral, −1.5 mm). Additionally, allow 5min to stand before removing to guarantee that the virous has completely dispersed. The establishment of MCAO operation occurred three days after injection.

Rats given the AAV-GFP vector were sacrificed three days after the injection to confirm the success of transfection. Brain sections (40 µm) were prepared using a Leica CM1850 cryostat (Leica Microsystems GmbH, Wetzlar, Germany), and the GFP-positive cell in the peri-ischemic area was observed using a fluorescence microscope (Nikaon, DSRi2, Japan). Three days following the injection, mice that had received the AAV-GFP vector were euthanized, and the amounts of Tmem30a mRNAs in the brain tissues were assessed by qRT-PCR.

**Bioinformatics analysis**

After QC of clean reads using Fast QC, we used Tophat2 software to map the high-quality reads to the rehMac8 reference genome. We then used the Cufflinks suite of tools to assemble transcriptome and quantify gene expression. Differentially expressed RNAs (DEmRNAs) in paired samples were obtained by R-package Limma. The screening criteria were as follows: 1) P value < 0.05; 2) The absolute value of log 2 (fold change) > 2. For differentially expressed genes, GO and KEGG pathway analysis was performed to investigate the significant enriched pathways (corrected P < 0.01) by R-package GO stats, and protein-protein interaction (PPI) network was obtained by STRING (<https://string-db.org/>).

**Quantitative real-time polymerase chain reaction (qRT-PCR) validation**

qRT-PCR was used to measure the quantities of variously expressed mRNAs in the brain tissues of the Macaca mulatta MCAO model. In summary, total RNA was reverse-transcribed into cDNA using the Thermo First cDNA Synthesis Kit (SinoGene, China) in compliance with the manufacturer's instructions. Gene expression was then evaluated by qRT-PCR using the 2×SG PCR MasterMix.

The following were the reaction conditions for qRT-PCR: 10 min at 95℃, 40 cycles of 20s at 95℃ and 30s at 60℃, followed by 15s at 95℃, 30s at 60℃, and 15s at 95℃. The 2-ΔΔCt method was used to calculate the relative expression levels after normalizing them to the internal β-actin.

The following primer pairs used: monkey *Tmem30a,* forward, 5’ ctggcacggtgctacctatt 3’ and reverse, 5’ Ttattgcagggactggaagg 3’; monkey ACTB, forward, 5’gatctggcaccacaccttct 3’ and reverse, 5’ggggtgttgaaggtctcaaa 3’; rat *Tmem30a,* forward, 5’CGTGAGATCGAGGGCAATGT 3’ and reverse, 5’ GGGTCTCCGTTTAACTGGCT 3’; rat GAPDH, forward, 5’ GTGCCAGCCTCGTCTCATAG 3’ and reverse, 5’AGAGAAGGCAGCCCTGGTAA 3’.

**Oxygen-glucose deprivation**

Following ten days of exposure to OGD/R, a hypoxic chamber (Billups Rothenberg, Inc., Del Mar, CA, USA) with 5% CO_2_, 0.02% O_2_, and 94.98% N_2_ was used to hold primary cortical neuron cultures at 37℃. The cultured medium was switched to glucose-free Dulbecco's Modified Eagle media (Gibco, Life Tech, Gaithersburg, MD, USA), and OGD for 2h. Then the neurons were re-cultured for a full day under normal circumstances.

**LDH measurements**

The degree of neuronal cell injury was assessed by utilizing the CytoTox 96 Non-Radioactive Cytotoxicity Assay kit (Promega) to measure the amount of lactate dehydrogenase (LDH) released into the culture medium. The assays were carried out in accordance with the manufacturer's guidelines. In a 96-well flat plate with a transparent bottom, 50 µL of the cultivated cell medium was taken and combined with 50 µL of CytoTox 96 reagent. At room temperature, the plate was incubated for 30 min. Absorbance was measured using a spectrophotometer equipped with an absorbance reader (Thermo Scientific, MA, USA) calibrated at 490 nm. The results for each sample were calculated by averaging the data from triplicate wells. To determine the cell damage index, the LDH release under each experimental condition was contrasted with the naive control..

**Overexpression of *Tmem30a* in vivo**

**Virus vector GV492-*Tmem30a* production**

Shanghai GeneChem supplied the recombinant lentivirus with the rat Tmem30a gene.

The gene of *Tmem30a* was amplified by PCR after designed primers. The primers of *Tmem30a* are: forward, 5’AGGTCGACTCTAGAG

GATCCCGCCACCATGGCGATGAACTATAGCGC 3’, reverse, 5’ TCCTTGTAG

TCCATACCAATGGTGATGTCAGCAGTATTAC 3’. Then GFP-expressing GV492 lentivirus vector inserted by BamHI / AgeI digestion to construct the plasmids GV492-*Tmem30a*. The clone obtained was confirmed by DNA sequencing. The lentivirus vectors were used for the transient transfection of 293T cells by liposome transfection method.

**Transfection of neuron in-vivo**

The neurons were used in the following experiments when primary neuron culture was stable at the fourth day. Neurons were transduced with LV- *Tmem30a* (multiplicity of infection, MOI: 300) lentivirus vectors. The titer of lentivirus vector LV- *Tmem30a* was 2.5 × 10^8^ transforming units (TU)/ml. Packaging and production of the GV492 was performed. The empty backbone vectors (CON335) were used as negative controls. Expression of *Tmem30a* protein in the cells was examined by Western blot after viral infection 72 h.

**Immunofluorescent**

Briefly, brain slices and cultured neurons were incubated at 4°C overnight with the following primary mouse antibodies in antibody diluent with background-reducing components: anti-Iba-1 1:200 (Wako, 016-26721); anti-NeuN 1:500 (millipore, MAB377); anti-GFAP1:500 (Santa, SC-33673); anti-MAP2 1:200 (Santa, SC-32791); anti-*Tmem30a* 1:200 (Abcam, ab217330); anti-pRIPK1(Ser 161) 1:200 (Affinity, AF7377); anti-pMLKL(Ser 358) 1:200 (Affinity, AF7420); and PSIVA 1:200 (Abcam, ab129817); The nuclear counterstain DAPI (VectaShield, Vector Laboratories) was used to counterstain the slides. For tissue imaging, filters for fluorescein isothiocyanate, Cy3, Texas Red, Cy5, and DAPI were employed in order to make it easier to unmix all colors later on based on their individual spectra.

**Western Blot**

Protein levels were measured using a BCA protein assay kit (Thermo Fisher Scientific, catalog # 23227) after brain tissue or cell lysates were collected in 1% NP40 buffer using the protease inhibitor cocktail (Roche, catalog # 4693159001) and phosphatase inhibitor PhosSTOP (Roche, catalog # 4906845001). Proteins were separated using SDS-PAGE according to their molecular weight, and they were subsequently wet transferred to a PVDF membrane at 70V for varied durations.

After being blocked in 5% milk in TBST for 1h, all membranes were probed overnight using the designated primary antibodies in antibody diluent at 4°C. Rabbit monoclonal anti-Tmem30a antibody (Abcam, 1:700), rabbit monoclonal anti-STAT5 antibody (Cell Signaling Technology, 1:1000), rabbit polyclonal anti-pSTAT5 antibody (Cell Signaling Technology, 1:200), mouse polyclonal anti-Bcl-2 antibody (Santa, 1:1000), mouse polyclonal anti-Bcl-xl antibody (Santa, 1:1000), rabbit polyclonal anti-caspase 3 antibody (Cell Signaling Technology, 1:1000), and mouse monoclonal anti-β-actin antibody (Zhongshanjinqiao, 1:1000) were among the primary antibodies. Using FluorChem Systems (Protein Simple), bands were created using the proper HRP-conjugated secondary antibodies (1:5000) and incubated on membranes in 5% milk for one hand. Image J was then used for analysis.

**TUNEL staining**

Apoptotic cells were demonstrated using TUNEL assay. The TUNEL Kit (Roche, catalog number 12156792910) was used. Slides with primary neurons with or without OGD therapy after LV infection or not were discarded according to the technique described by the manufacturer.

**Supplemental Figures**


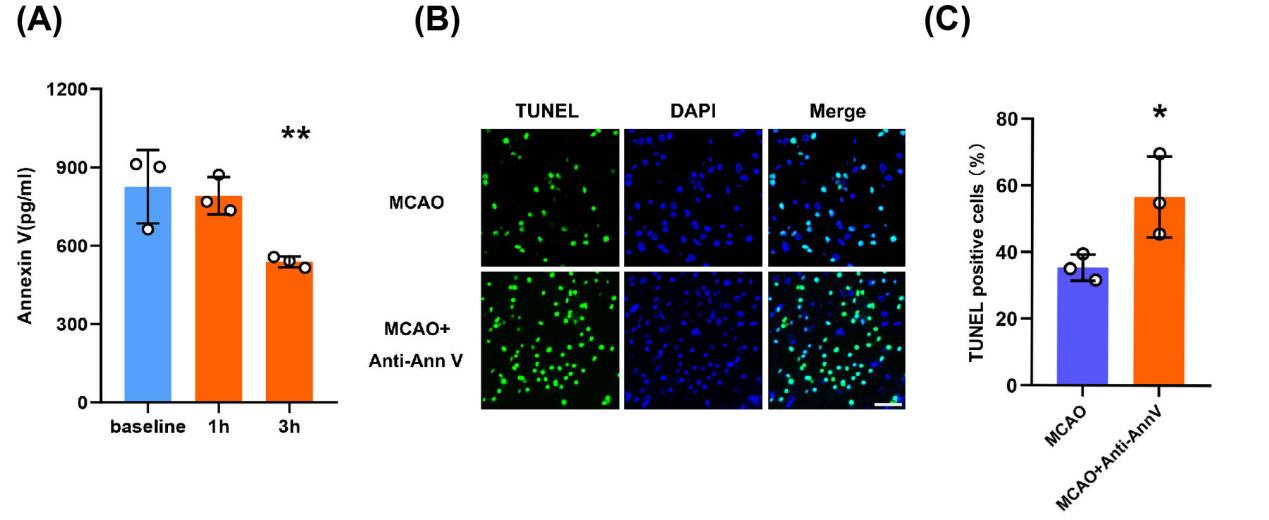


**Figure S1. Administrating anti-Annexin V reduces plasma Annexin V levels and increases the Tunnel-positive cells after tMCAO (transient MCA occlusion).** (A) The concentration of plasma Annexin V before and after administration of anti- Annexin V antibody for 1 and 3 h in normal C57 mice (n=3). (B) Representative images of cell death based on TUNEL assay in the ischemic ipsilateral brain regions of mice 24 h after 60 min tMCAO (transient MCA occlusion) (n=3) Bar=20 µm. (C) Quantification of neuronal death based on a neural marker NeuN and TUNEL assay in the ischemic ipsilateral brain regions of mice 24 h after 60 min tMCAO (transient MCA occlusion) (n=3). ** *p* < 0.01 by one-way ANOVA with Bonferroni’s multiple comparison in A. * *p* < 0.05, two-tailed t test in C.


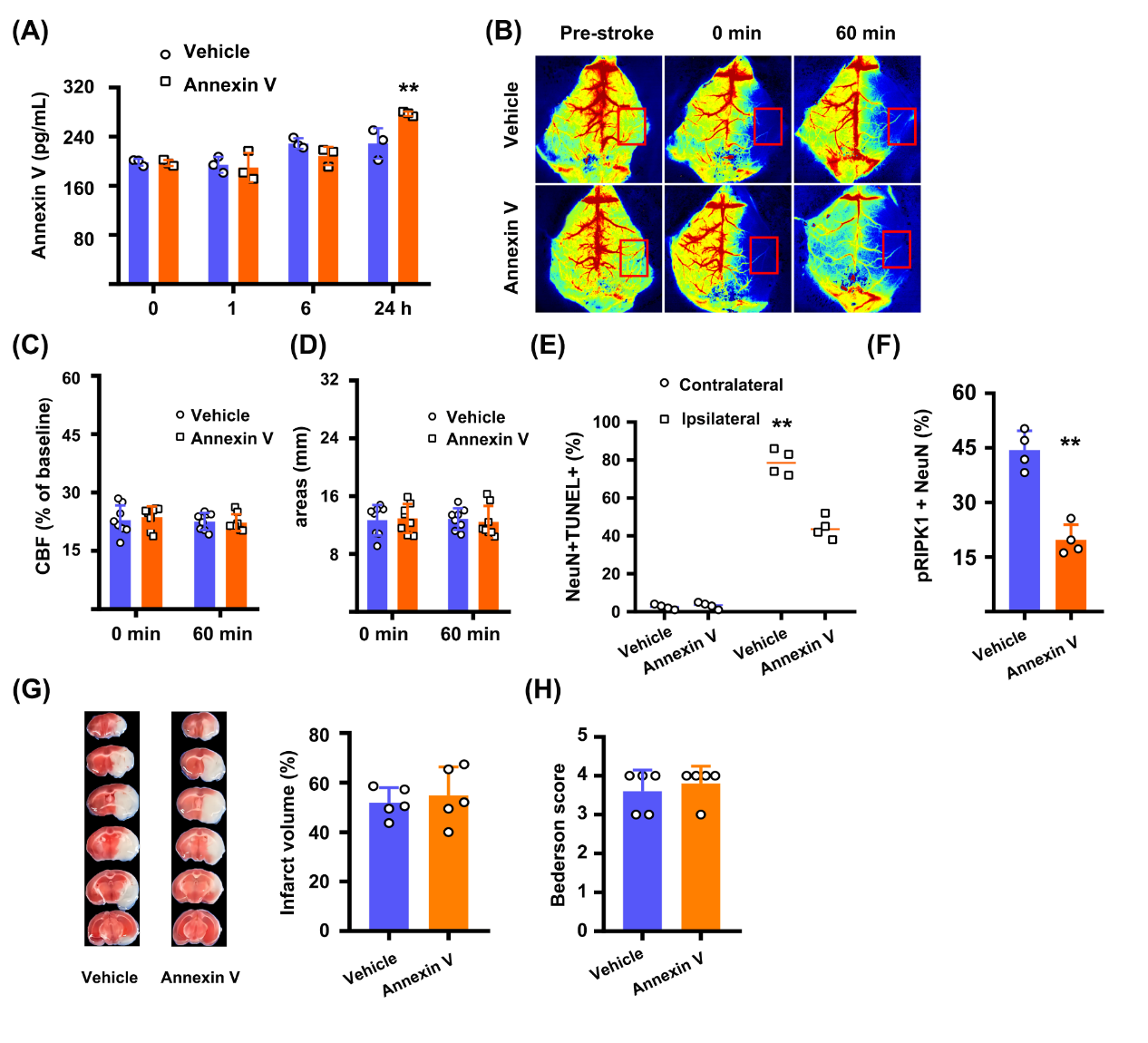


**Figure S2. Administrating Annexin V reduces brain damage in stroke model of mice.**

Annexin V levels in brain tissue in WT mice (n=3). (B) Representative LSI images showing cortical perfusion from mice receiving Annexin V or PBS at different time points in tMCAO (transient MCA occlusion) mice (n=8). Rectangular area used for quantifying data from four independent experiments or mice per group in C and D. (C) LSI recordings for penumbra ROI between PBS and Annexin V in tMCAO (transient MCA occlusion) mice (n=8). (D) Penumbra areas between PBS and Annexin V in tMCAO (transient MCA occlusion) mice (n=8). (E) Quantification of neuronal death based on a neural marker NeuN and TUNEL assay in the ischemic ipsilateral brain regions of mice 24 h after 60 min tMCAO (transient MCA occlusion) (n=4). (F) Quantification of neuronal death based on a neural marker NeuN and necroptosis marker pRIPK1 in the ischemic ipsilateral brain regions of mice 24 h after 60 min tMCAO (transient MCA occlusion) (n=4). (G) Representative TTC-stained brain sections and infarct volumes 24 h after pMCAO (permanent MCA occlusion) (n=5). (H) Neurological score in mice 1 day after stroke (n= 5). ** *p* < 0.01 by one-way ANOVA with Bonferroni’s multiple comparison (B, C, E) and by two-tailed t test (D and F).


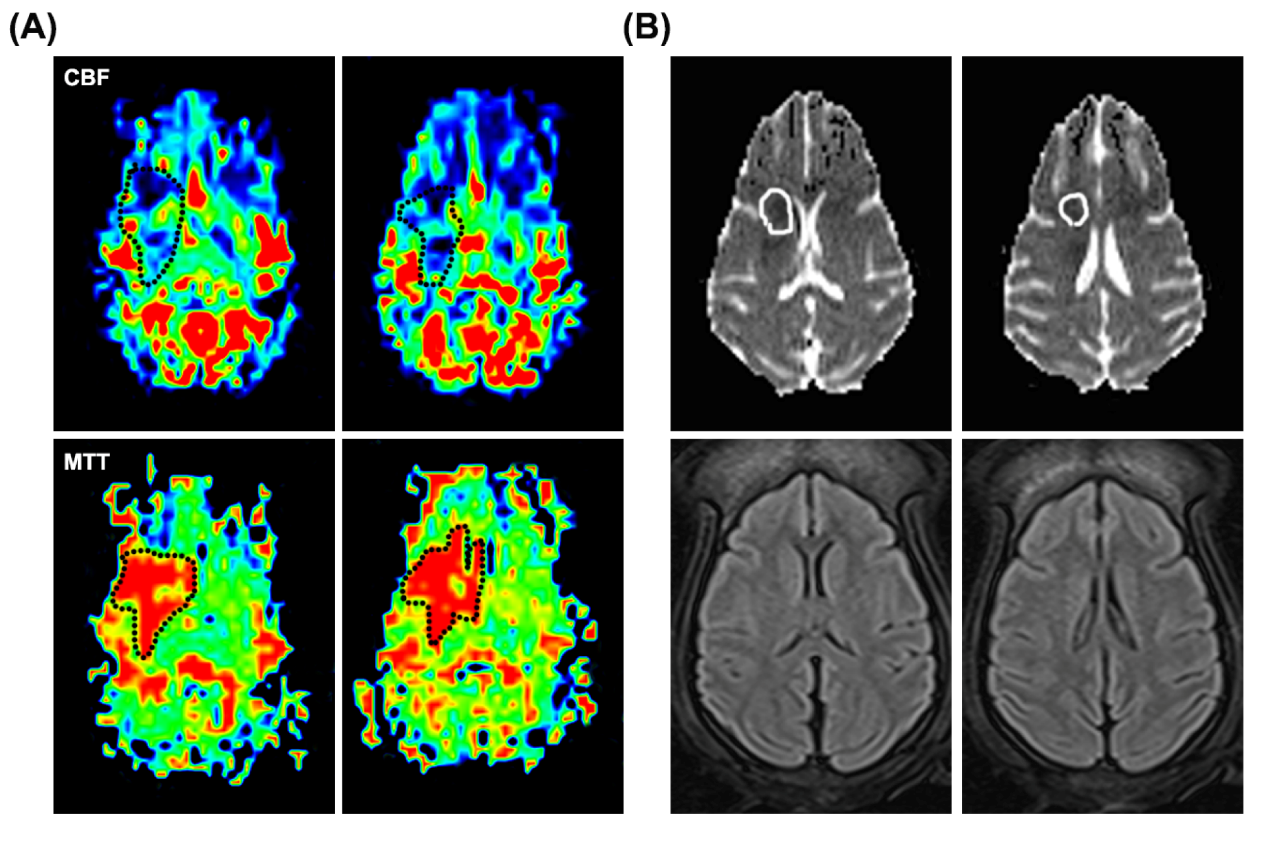


**Figure S3. MRI images reveals a similar perfusion deficit area from the ischemic stroke model.**

(A) CBF images (upper) and MTT images (down) 3 h after stroke onset. (B) ADC images (upper) and Flair-T2 images (down) 3 h after stroke onset.


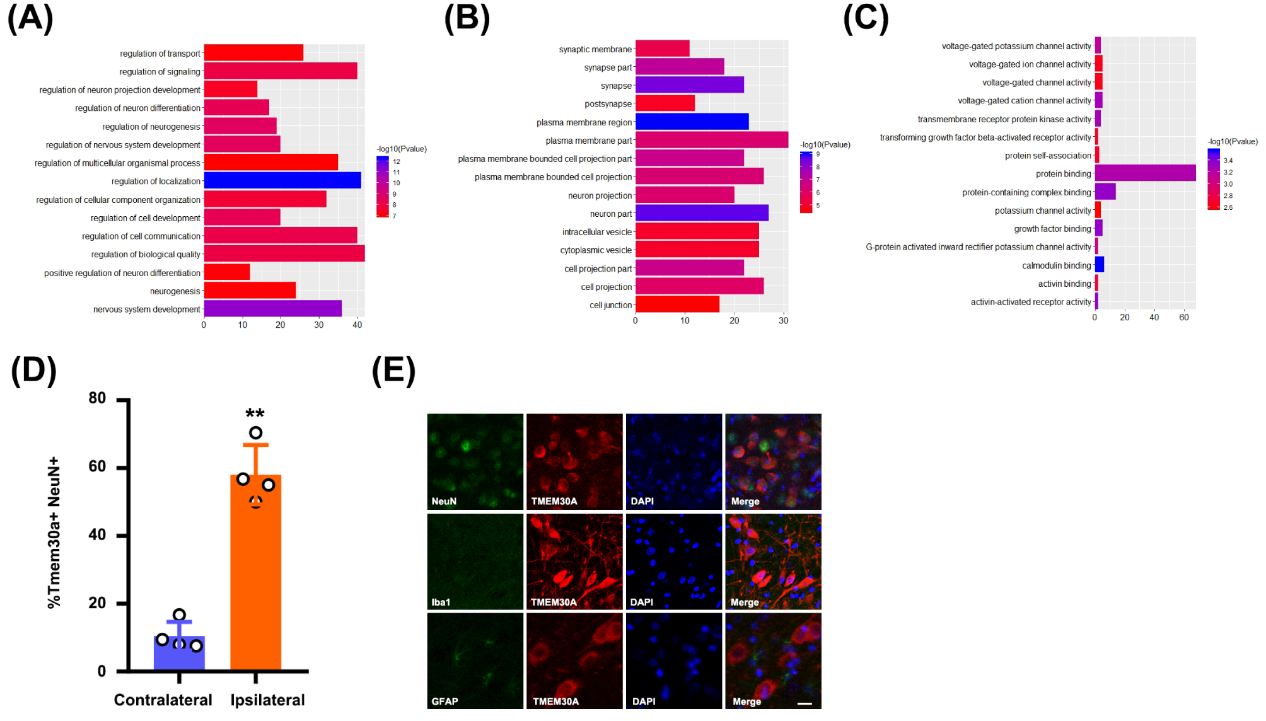


**Figure S4. *Tmem30a* is upregulated in neurons within the penumbra tissues.**

(A) Biological Process (BP) analysis indicated regulation of biological quality, location, and signaling as the top 3 most significant factors. (B) protein binding as the most significant factor in Molecular Function (MF) analysis. (C) plasma part, neuron part, and cell projection as top 3 in Cellular Component (CC) analysis. (D) Quantification of *Tmem30a* positive neuronal in NeuN positive neurons in the ipsilateral penumbra region and that in the contralateral region in rat tMCAO (transient MCA occlusion) models (n=4). (E) Immunofluorescence staining of *Tmem30a* and typical markers (NeuN for neurons, GFAP for astrocytes, and Iba1 for microglia) of brain cells in the cerebral penumbra cortex in rat tMCAO (transient MCA occlusion) models. Bar=50 µm. ** *p* < 0.01, two-tailed t test in D.

、
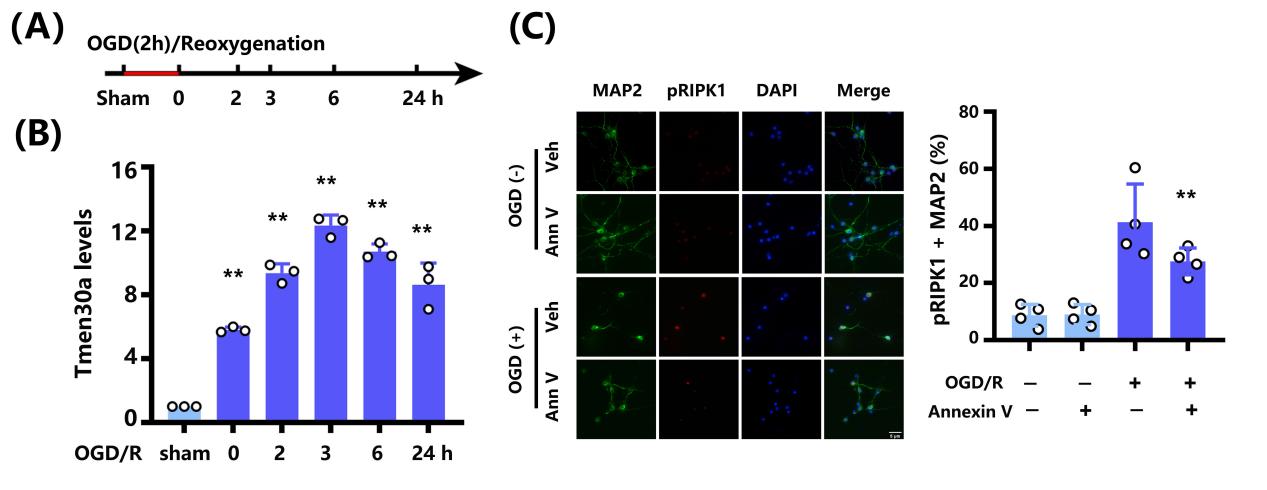


**Figure S5. Upregulation of *Tmem30a* in neuron mitigates pRIPK1 expression.**

(A) Diagram of 2h OGD and various duration of reoxygenation for neuron. (B) Tmem30a levels after OGD and reoxygenation (n=3). (C) Representative images and quantification of pRIPK1 positive cells in primary neuron undergoing 2h OGD and 24h reoxygenation

(n= 4). Bar=5μm. ** *p* < 0.01, by one-way ANOVA with Bonferroni’s multiple comparison in B and C.


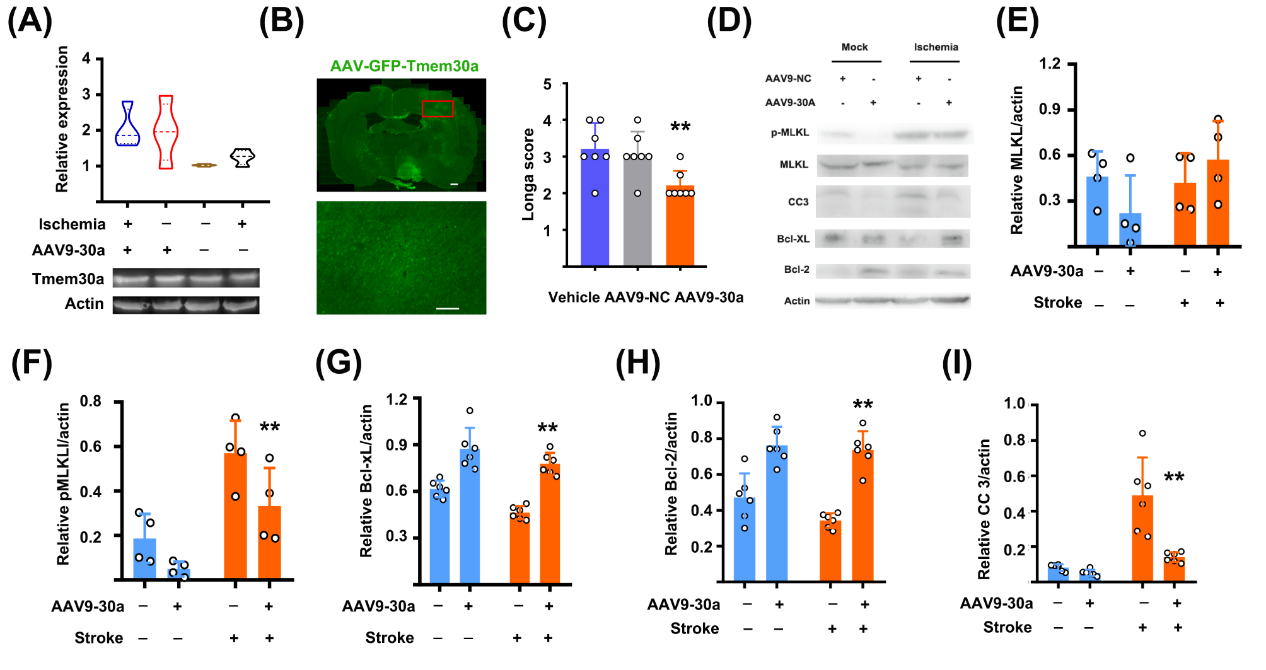


**Figure S6. AAV9-Tmem30a reduced apoptosis and necroptosis in rat after stroke.**

(A) Bar graphs represent expression of *Tmem30a* in cortex at 4 days post injection in naive and post-stroke tissue (n=7). (B) AAV9/Tmem30a-GFP construct was injected into the cortex and Tmem30a expression (Green) was increased in the right side. Bar=100μm. (C) Longa score in rats receiving AAV9-Tmem30a or AAV9-NC after stroke (n=7). (D) Western blot analysis of the protein levels of MLKL, pMLKL, Bcl-XL, Bcl-2, and cleaved-caspase3 in *Tmem30a* overexpression penumbra tissues 24 h after stroke (n=4). (E) Bar graph depicting MLKL protein levels in penumbra tissues 24 h after stroke. (F) Bar graph depicting pMLKL protein levels in penumbra tissues 24 h after stroke. (G) Bar graph depicting Bcl-xl protein levels in penumbra tissues 24 h after stroke. (H) Bar graph depicting Bcl-2 protein levels in penumbra tissues 24 h after stroke. (I) Bar graph depicting cleaved-caspase3 protein levels in penumbra tissues 24 h after stroke. ***p* < 0.01, by one-way ANOVA with Bonferroni’s multiple comparison in (A-I).


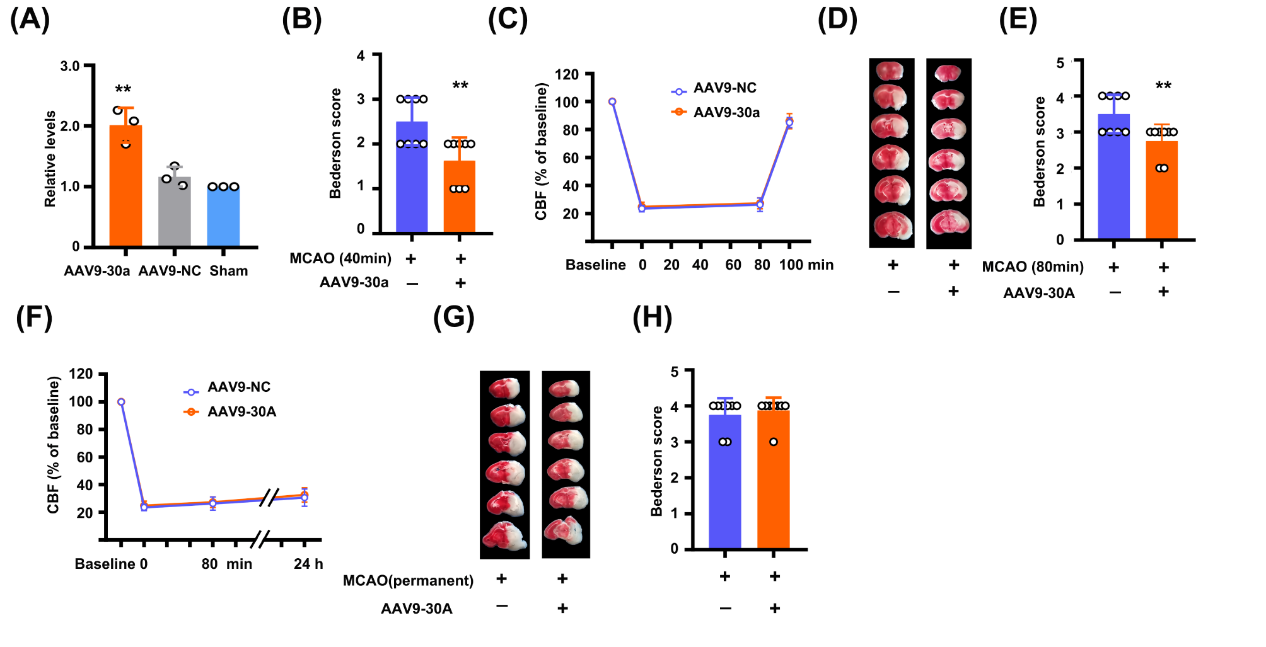


**Figure S7. AAV9-*Tmem30a* reduced ischemic damages in mice after stroke.**

(A) Relative *Tmem30a* levels in mice with AAV9-*Tmem30a* and AAV9-NC in normal mice (n=3). (B) Quantification of Bederson scores 24 h after 40 min tMCAO (transient MCA occlusion) in C57BL/6 mice. (C) CBF levels during 80 min tMCAO in C57BL/6 mice receiving AAV9-*Tmem30a* or AAV9-NC (n=8). (D) Representative images of TTC staining in brain sections 24 h after 80-min tMCAO (transient MCA occlusion) in C57BL/6 mice. (E) Quantification of Bederson scores 24 h after 80 min tMCAO (transient MCA occlusion) in C57 BL/6 mice. (F) CBF levels in permanent models of C57BL/6 mice receiving AAV9-*Tmem30a* or AAV9-NC (n=8). (G) Representative images of TTC staining in brain sections 24 h after permanent MCAO (n=8). (H) Quantification of Bederson scores 24 h after permanent MCAO. ** p < 0.01, by one-way ANOVA with Bonferroni’s multiple comparison (A) and two-tailed t test (B and E).


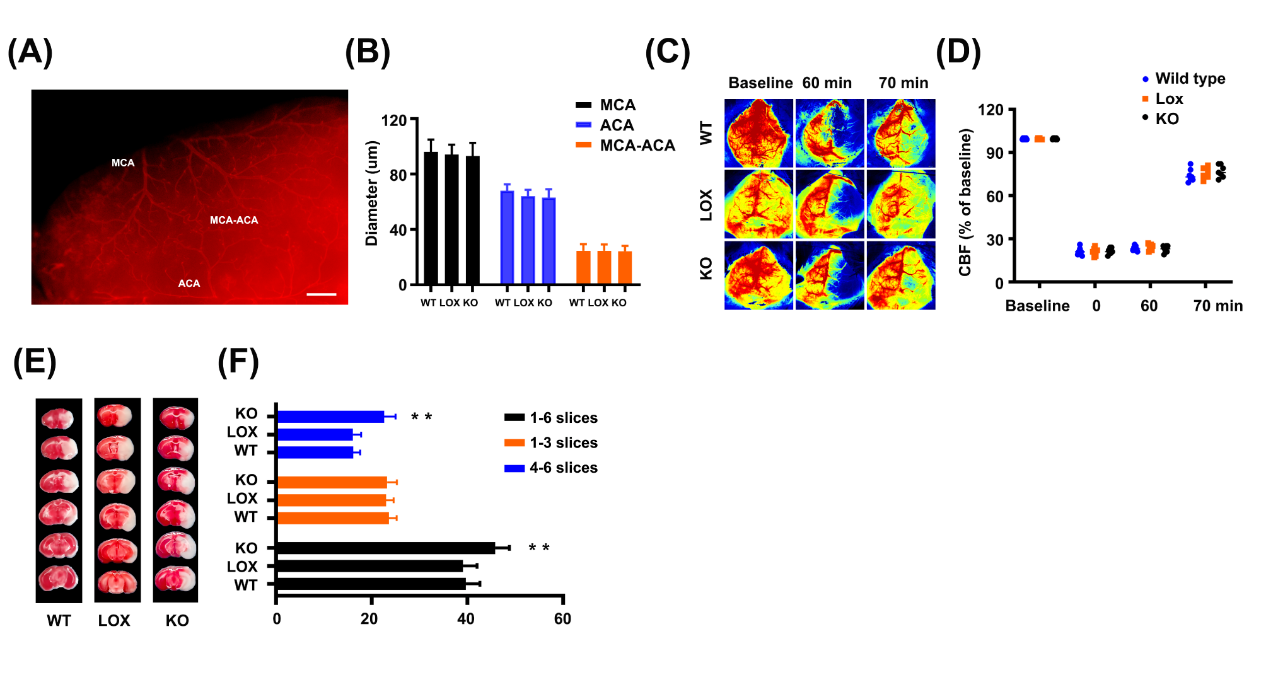


**Figure S8. Brain arteries, CBF, and histological characteristics among wild- type (WT), Tmem30a^loxP/loxP^ (Lox), and Tmem30a FnKO (KO) mice.**

(A) The gross anatomy of the MCA territory as shown by vascular infusion of latex mixed with carbon black. Bar= 150μm. (B) Diameters in MCA, ACA, and collateral between MCA and ACA among three groups (n=3). (C) Representative LSI images for CBF measurements during focal ischemia. Rectangular area used for quantifying data from three independent experiments or mice per group in D. (D) Bar graph depicting CBF levels during tMCAO (transient MCA occlusion) procedures (n=7). (E) Representative TTC staining of brain slices 24 h after stroke onset. (F) Infarct sizes among three groups, including 1-3 brain slices, 4-6 slices, and the total (n=8 or n=9). ***p* < 0.01 by one-way ANOVA with Bonferroni’s multiple comparison in F.

*
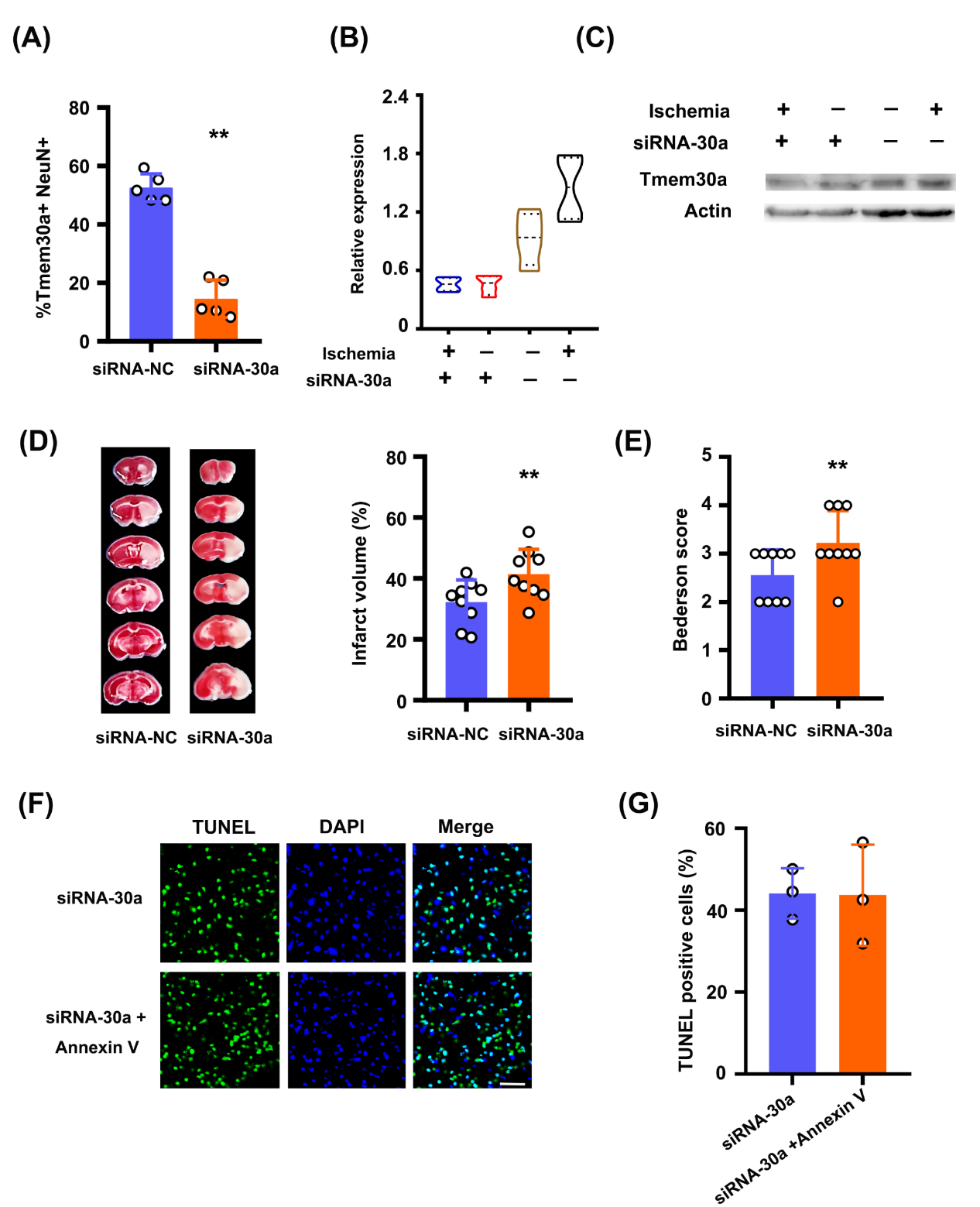
*

**Figure S9. siRNA-Tmem30a pre-injection led to a worse stroke outcome in mice after tMCAO (transient MCA occlusion).**

1. Percentage of Tmem30a positive neurons in NeuN positive neurons in the penumbra regions from tMCAO (transient MCA occlusion) mice receiving siRNA-NC or siRNA-Tmem30a (n=5). (B-C) Quantification and Western blot analysis of Tmem30a levels in cortex at 3 days post injection in naive and post-stroke tissue (n=4). (D) Representative images of TTC staining and infarct volumes in brain sections 24 h after 60-min tMCAO in C57BL/6 mice receiving siRNA-NC or siRNA-Tmem30a (n=9). (E) Bederson scores 1 day after stroke in mice receiving siRNA-Tmem30a or siRNA-NC (n=9). (F) Representative images of cell death based on TUNEL assay in the ischemic ipsilateral brain regions of siRNA-Tmem30a mouse tMCAO (transient MCA occlusion) models with or without Annexin V (n=3) Bar=20 µm. (G) Quantification of neuronal death based on a neural marker NeuN and TUNEL assay in the ischemic ipsilateral brain regions of siRNA-Tmem30a mouse tMCAO (transient MCA occlusion) models with or without Annexin V (n=3). ***p* < 0.01, two-tailed t test (A, D and E).

**
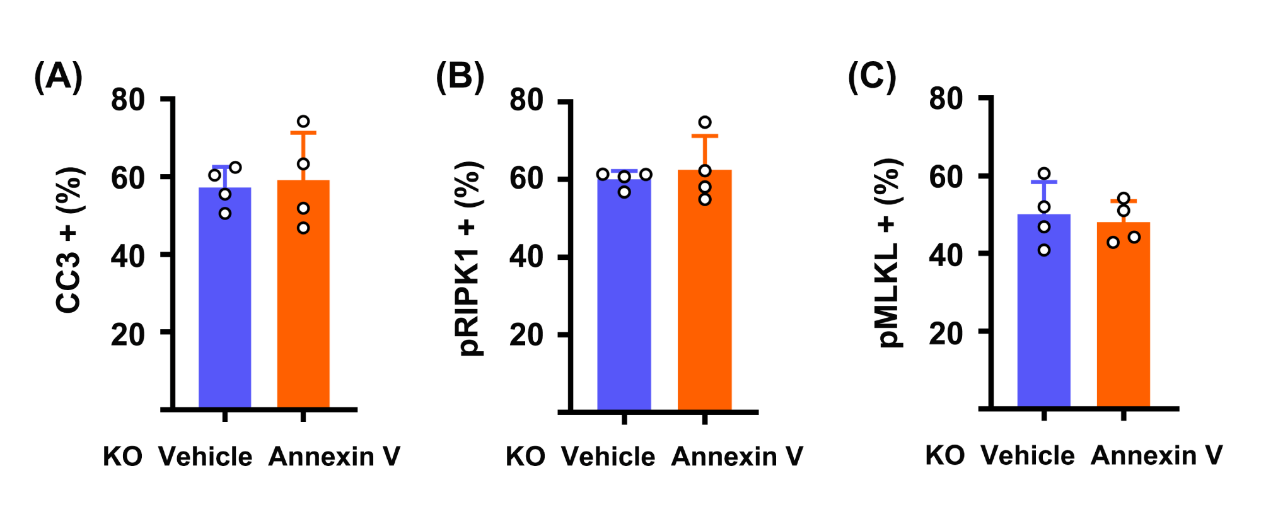
**

**Figure S10. CC3^+^, pMLKL^+^, and pRIPK1^+^ neurons in Tmem30a FnKO mice after Annexin V administration.**

(A). Quantification of CC3 positive neuron in penumbra regions 24 h after 60 min tMCAO (transient MCA occlusion) in Tmem30a FnKO mice (n=4). (B). Quantification of pRIPK1 positive neuron in penumbra regions 24 h after 60 min tMCAO (transient MCA occlusion) in Tmem30a FnKO mice (n=4). (C). Quantification of pMLKL positive neuron in penumbra regions 24 h after 60 min tMCAO (transient MCA occlusion) in Tmem30a FnKO mice (n=4)

**Supplementary Table**

**Table S1** ANXA5 mRNA expression in cardioembolic stroke samples and normal control samples

| ID | adj.P.Val | P.Value | t | B | logFC | Gene.symbol | Gene.title | Time | FC |
| --- | --- | --- | --- | --- | --- | --- | --- | --- | --- |
| 200782_at | 6.98E-08 | 6.98E-08 | -6.3300828 | 7.940856 | 0.60895739 | ANXA5 | annexin A5 | 3h | 1.525156607 |
| 200782_at | 2.56E-06 | 2.56E-06 | -5.3114066 | 4.3937182 | 0.57032174 | ANXA5 | annexin A5 | 5h | 1.484854676 |
| 200782_at | 1.73E-09 | 1.73E-09 | -7.3549515 | 11.518071 | 0.7771783 | ANXA5 | annexin A5 | 24h | 1.713775699 |

**Table S2** Baseline demographic and clinical characteristics of patients and healthy controls

|  | Stroke patients | Healthy controls | p Value |
| --- | --- | --- | --- |
|  | （N=22） | (N=22) |  |
| Age, y | 64.45 ± 8.66 | 61.91 ± 9.22 | 0.61 |
| Women, n (%) | 10 (45.45) | 11 (50.00) | 0.76 |
| Vascular risk factors, n (%) |  |  |  |
| Hypertension | 9 (40.90) | 7 (31.82) | 0.53 |
| Diabetes mellitus | 6 (27.27) | 7 (31.82) | 0.74 |
| Atrial fibrillation | 3 (13.64) | 2 (9.09) | 0.64 |
| Current smoking | 9 (40.90) | 6 (27.27) | 0.34 |
| Clinical findings |  |  |  |
| Systolic blood pressure, mm Hg | 154 ± 17 | 138 ± 13 | 0.13 |
| Admission NIHSS score | 11 (9-14) | — | — |
| From stroke onset to blood collection | 188 (160-214) | — | — |

**Table S3** Baseline demographic and clinical characteristics of patients

|  | Total  （N=60） | ENI +  (N=26) | ENI -  (N=34) | p Value |
| --- | --- | --- | --- | --- |
| Age, y | 64.15 ± 9.14 | 64.54 ± 8.32 | 63.85 ± 9.84 | 0.48 |
| Women, n (%) | 25 (41.67) | 14 (53.85) | 11 (32.35) | 0.56 |
| Prior vascular risk factors, n (%)  Hypertension  Diabetes mellitus  Atrial fibrillation  Current smoking | 34 (56.67)  10 (18.33)  23 (38.33)  11 (18.33) | 15 (57.69)  4 (15.38)  9 (34.62)  4 (15.38) | 19 (55.88)  6 (17.65)  14 (41.18)  7 (20.59) | 0.54  0.55  0.40  0.43 |
| Clinical findings  Systolic blood pressure, mm Hg  Admission NIHSS score  NIHSS score at 24 h  Patients with penumbra at baseline, n (%) | 146 ± 18  17.5 (12.25-26)  10 (6-16.75)  41 (68.33) | 144 ± 17  19 (12-26)  6.5 (2.75-10)  22 (84.62) | 149 ± 19  16.5 (12.8-25.5)  16 (9.5-22.5)  19 (55.88) | 0.64  0.79  0.001  0.017 |
| Intravenous thrombolysis | 56 (93.33) | 25 (96.15) | 31 (91.18) | 0.41 |
| Workflow times  From stroke onset to blood collection  From stroke onset to groin puncture  From stroke onset to revascularization | 156 (132-186)  175 (153-200)  225 (193-256) | 151 (126-182)  169 (139-203)  217 (186-236) | 158 (143-186)  176 (159-199)  245 (204-271) | 0.40  0.43  0.10 |

**Table S4** Case-control gene expression analysis

| Experiment type | Expression profiling by array |
| --- | --- |
| Summary | Blood from subjects with cardioembolic stroke and controls was collected, and the RNA extracted was interrogated and whole genome U133 Affymetrix Arrays. Twenty-three control samples and sixty-nine cardioembolic stroke samples were assayed. |
| Overall design | Blood from subjects with cardioembolic stroke and controls was collected, and the RNA extracted was interrogated and whole genome U133 Affymetrix Arrays. Twenty-three control samples and sixty-nine cardioembolic stroke samples were assayed. Cardioembolic stroke subjects were analyzed at three time points: less than 3 h, 5 h, and 24 h following the event. |

**References**

54. Jadhav AP, Aghaebrahim A, Jankowitz BT, Haussen DC, Budzik RF, Bonafe A, et al. Benefit of Endovascular Thrombectomy by Mode of Onset: Secondary Analysis of the DAWN Trial. Stroke. 2019;50(11):3141-6.

55. Tsien JZ, Chen DF, Gerber D, Tom C, Mercer EH, Anderson DJ, et al. Subregion- and cell type-restricted gene knockout in mouse brain. Cell. 1996;87(7):1317-26.

56. Liu X, Wen S, Zhao S, Yan F, Zhao S, Wu D, et al. Mild Therapeutic Hypothermia Protects the Brain from Ischemia/Reperfusion Injury through Upregulation of iASPP. Aging Dis. 2018;9(3):401-11.

57. Esposito E, Li W, E TM, Park JH, Şencan I, Guo S, et al. Potential circadian effects on translational failure for neuroprotection. Nature. 2020;582(7812):395-8.

58. El Amki M, Glück C, Binder N, Middleham W, Wyss MT, Weiss T, et al. Neutrophils Obstructing Brain Capillaries Are a Major Cause of No-Reflow in Ischemic Stroke. Cell Rep. 2020;33(2):108260.

59. Wang L, Wu L, Duan Y, Xu S, Yang Y, Yin J, et al. Phenotype Shifting in Astrocytes Account for Benefits of Intra-Arterial Selective Cooling Infusion in Hypertensive Rats of Ischemic Stroke. Neurotherapeutics. 2022;19(1):386-98.

60. Duan Y, Wu D, Huber M, Shi J, An H, Wei W, et al. New Endovascular Approach for Hypothermia With Intrajugular Cooling and Neuroprotective Effect in Ischemic Stroke. Stroke. 2020;51(2):628-36.

61. Li M, Li Z, Yao Y, Jin WN, Wood K, Liu Q, et al. Astrocyte-derived interleukin-15 exacerbates ischemic brain injury via propagation of cellular immunity. Proc Natl Acad Sci U S A. 2017;114(3):E396-e405.

62. Clarkson AN, Huang BS, Macisaac SE, Mody I, and Carmichael ST. Reducing excessive GABA-mediated tonic inhibition promotes functional recovery after stroke. Nature. 2010;468(7321):305-9.
